# Effective therapeutic targeting of tumor lineage plasticity in neuroendocrine prostate cancer by BRD4 inhibitors

**DOI:** 10.1016/j.apsb.2025.01.007

**Published:** 2025-01-22

**Authors:** Xiong Zhang, Yatian Yang, Hongye Zou, Yang Yang, Xingling Zheng, Eva Corey, Amina Zoubeidi, Nicolas Mitsiades, Ai-Ming Yu, Yuanpei Li, Hong-Wu Chen

**Affiliations:** a Department of Biochemistry and Molecular Medicine, School of Medicine, University of California Davis, Sacramento, CA 95817, USA; b Department of Urology, University of Washington, Seattle, DC 98915, USA; c Department of Urologic Sciences, University of British Columbia, Vancouver, BC V5Z1M9, Canada; d Department of Internal Medicine, Division of Hematology and Oncology, School of Medicine, University of California Davis, Sacramento, CA 95817, USA; e Comprehensive Cancer Center, University of California Davis, Sacramento, CA 95817, USA; f VA Northern California Health Care System-Mather, Mather, CA 95655, USA

**Keywords:** Tumor lineage plasticity, BRD4, Neurogenesis, AZD5153, FOXA1, ChIP-seq, PDX, BRN2

## Abstract

Tumor lineage plasticity (LP) is an emerging hallmark of cancer progression. Through pharmacologically probing the function of epigenetic regulators in prostate cancer cells and organoids, we identified bromodomain protein BRD4 as a crucial player. Integrated ChIP-seq and RNA-seq analysis of tumors revealed, for the first time, that BRD4 directly activates hundreds of genes in the LP programs which include neurogenesis, axonogenesis, EMT and stem cells and key drivers such as *POU3F2* (BRN2), *ASCL1/2*, *NeuroD1*, *SOX2/9*, *RUNX1/2* and *DLL3*. Interestingly, BRD4 genome occupancy is reprogrammed by anti-AR drugs from facilitating AR function in CRPC cells to activating the LP programs and is facilitated by pioneer factor FOXA1. Significantly, we demonstrated that BRD4 inhibitor AZD5153, currently at clinical development, possesses potent activities in complete blockade of tumor growth of both *de novo* neuroendocrine prostate cancer (NEPC) and treatment-induced NEPC PDXs and that suppression of tumor expression of LP programs through reduction of local chromatin accessibility is the primary mechanism of action (MOA) by AZD5153. Together, our study revealed that BRD4 plays a fundamental role in direct activation of tumor LP programs and that its inhibitor AZD5153 is highly promising in effective treatment of the lethal forms of the diseases.

## Graphical abstract

This study demonstrated that BRD4 plays a fundamental role in direct activation of tumor lineage plasticity programs and that its inhibitor AZD5153 is highly promising in effective treatment of the lethal forms of prostate cancer.

**Figure:**
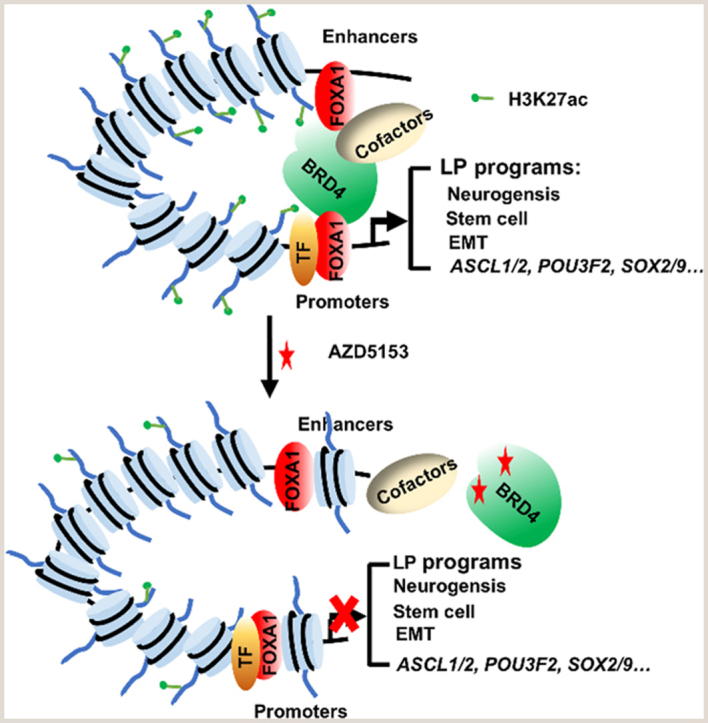

## 1 Introduction

Tumor lineage plasticity (LP) is an emerging hallmark of cancer progression. It is characterized by expression of distinct gene programs that have similarities to those in the early developmental cell lineages^1^^,^^2^. LP represents a major mechanism of treatment resistance and disease relapse^3^^,^^4^. The vast majority of prostate cancer (PCa) tumors are androgen receptor (AR) positive adenocarcinomas at initial diagnosis^5^. Thus, AR pathway inhibitors (ARPIs) are widely used for treatment of PCa^6^. Although treatment with ARPIs significantly increases the survival time of patients with advanced PCa, tumors in most patients will ultimately develop resistance to these treatments^7^. Tumor cells often transdifferentiate from luminal cell type to more lethal types with small cell features, expressing neuronal transcriptional factors and markers^8^^,^^9^. Such disease progression is often referred to as treatment-induced neuroendocrine prostate cancer (t-NEPC), which accounts for 20%–30% of treatment-refractory, castration-resistant prostate cancer (CRPC)^10^. *De novo* NEPC is rare; it accounts less than 1% of all PCa cases^11^. Currently, no effective treatments are available for NEPCs^12^.

Studies show that t-NEPCs and *de novo* NEPCs share similar genomic and epigenetic landscapes, and that transcriptional factors (TFs), such as BRN2, SOX2 and ASCL1 are the common drivers for the NEPC diseases progression^13^, ^14^, ^15^. They play crucial roles in the transdifferentiation from CRPC adenocarcinomas to NEPCs through activating LP gene programs which include neurogenesis, axonogenesis, stem cell and epithelial mesenchymal transition (EMT)^13^, ^14^, ^15^. However, the mechanism of LP development is poorly understood. Epigenetic reprograming and dysregulated transcriptional networks are strongly implicated in the activation of LP programs^16^. TFs such as BRN2 and ASCL1 are demonstrated to play vital roles in the development of NEPC^13^, ^14^, ^15^. However, those drivers are considered undruggable. Except that several studies documented the function of EZH2 in promoting NEPC progression, the role of other epigenetic regulators in NEPC has not been well reported^17^, ^18^, ^19^, ^20^. Thus, there is still an urgent need to develop effective treatment for NEPCs.

Epigenetic regulator BRD4 (bromodomain-containing protein 4) belongs to bromodomains and extraterminal (BET) protein family^21^. BRD4 contains two conserved N-terminal bromodomains (BD1 and BD2) that are essential for recognition of acetylated histone and non-histone proteins, and recruitment of TFs to activate gene expression^22^. BRD4 may also possess chromatin remodeling and histone acetylation activities^23^. It plays important roles in cell growth, differentiation as well as metabolism and DNA repair^24^. Many oncogenes including *MYC*, *RUNX1* and *FOSL*, are directly activated by BRD4^25^. *BRD4* is often overexpressed in many cancers thus making BRD4 as an attractive therapeutic target^25^. In prostate adenocarcinomas, BRD4 associates with AR and is recruited to AR target genes, thus activating AR signaling pathways for growth and survival of adenocarcinoma cells^26^. Recently, a study reported that BRD4 functions with E2F1 to regulate cell cycle associated programs and that BRD4 inhibitors decrease growth of tumor models^27^. However, the functional mechanisms of BRD4 in NEPCs, such as its directly controlled gene programs are still poorly understood.

Several first-generation inhibitors of BRD4, *e.g.*, JQ1, iBET762, and OTX015, have been developed and displayed promising antitumor activity in preclinical models^28^. AZD5153 is a bivalent inhibitor of BET bromodomains. It binds to both BD1 and BD2^29^. Compared to first-generation BET inhibitors such as JQ1, AZD5153 exhibited improved potency both *in vitro* and *in vivo*^30^. Phase I and Phase II clinical trials of AZD5153 in patients with relapsed or refractory solid tumors including adenocarcinoma of prostate cancer (NCT03205176) demonstrated that AZD5153 has a relatively safe profile^31^. However, whether AZD5153 is effective in treating NEPCs is unclear.

Through integrated functional genomics studies, we found that BRD4 drives prostate cancer progression to NEPC by directly activating LP gene programs. Further mechanistic analysis revealed that BRD4 stimulates LP gene expression by increasing local chromatin accessibility. Interestingly, we also found that the functions of BRD4 are facilitated by pioneer factor FOXA1. Importantly, we demonstrated that BRD4 inhibitor AZD5153 possesses potent activities in blocking tumor growth of both *de novo* NEPC and t-NEPC.

## 2 Materials and methods

### 2.1 Organoid development and viability assay

Organoid development was established as described^32^ with some modifications. Briefly, fresh PDX tissue samples were placed in Advanced DMEM/F12 medium (Gibico, 12634010) in cell culture dish and washed twice before being minced with razor blade into fragments of <1 mm^3^. Dissected tissues were enzymatically digested in the medium in 50 mL Conical tubes containing 2 mg/mL collagenase type IV (sigma, C4-BIOC) and 100 μg/mL DNase I (sigma, DN25) at 37 °C on a shaker set to 220 rpm for 90 min. After centrifugation at 4 °C at 1000 rpm for 2 min, pelleted cell clusters were resuspended in 1 × RBC buffer (ThermoFisher, 00-4333-57) and incubated for 2 min on ice to remove red blood cells. The pellet was then washed and resuspended with the medium at a density of 5 × 10^6^ cells/mL. The resuspended cells were mixed with growth factor reduced Matrigel (Corning, 354230) in a 1:4 volume ratio and seeded into 96-well plates at a volume of 30 μL/well (3 × 10^4^ cells per well). The 96-well plates were incubated at 37 °C for 30 min to solidify the Matrigel before adding 100 μL prostate-specific growth medium each well. The prostate-specific growth medium for organoids was advanced DMEM/F12 supplemented with 1 × GlutaMAX (ThermoFisher, 35050061), 1 × Pen Strep (ThermoFisher, 15070063), 1 × B27 (Invitrogen, A3582801), 1.25 mmol/L *N*-acetylcysteine (Sigma, A0737), 50 ng/mL epidermal growth factor (sigma, E5036), 20 ng/mL human recombinant FGF-10 (Peprotech, AF-100-26), 10 ng/mL recombinant human FGF-basic (Peprotech, 100-18B), 500 nmol/L A-83-01 (Sigma, SML0788), 10 μmol/L SB202190 (Sigma, S7067), 10 mmol/L Nicotinamide (Sigma, N0636), 1 nmol/L Dihydrotestosterone (Sigma, D073), 1 μmol/L PGE2 (Sigma, P5640), 50 ng/mL Noggin (Peprotech, 120-10C) and 5% *R*-spondin conditioned media from the 293T cell culture. After 7 days of incubation, the organoids were treated as indicated and then incubated for an additional 4 days. For organoid viability, Cell-Titer Glo reagents (Promega, G9243) were added, and luminescence was measured by GLOMAX microplate luminometer (Promega), according to the manufacturer's instructions. All experimental points were set up as triplicates as biological replication. The data are presented as percentage of viable cells with vehicle-treated cells set as 100. For immunofluorescence assay, 100 μL of diluted live/dead reagents (Thermofisher, L34970) was added to each well, followed by 30 min of incubation at 37 °C. Fluorescence microscope was used to capture images of calcein AM (494 nm/517 nm, green) to visualize the live cells, and of ethidium bromide homodimer-1 (528/617 nm, red) to identify the dead cells. The above assays were performed in triplicates. The entire experiments were repeated three times.

### 2.2 Cryosectioning and immunofluorescence (IF)

Fresh samples of prostate PDX tissues were cut into pieces with a diameter of 0.5 cm and snap frozen using Tissue-OCT Compound (Sakura Finetek, MPSMK-981385). OCT-embedded tissues were sectioned into pieces with a thickness of 5 μm using a cryostat (Leica CM 1950, Netherlands) at −20 °C. The slides were fixed with 4% paraformaldehyde solution (ThermoFisher, J19943.K2), permeabilized with permeabilization buffer (2% Triton™ X-100 in PBS) and blocked by blocking buffer (10% FBS in PBS). The slides were then incubated with primary antibodies overnight at 4 °C followed by incubation with fluorescently labeled secondary antibody dilutions at room temperature for 1 h Hoechst33342 (ThermoFish, H3570) were used to label the nuclei. Images were acquired using Zeiss LSM780 Confocal harboring 405-, 488-, 561-, and 633-nm lasers and analyzed using ImageJ software. The antibodies and dilution ratio used are shown in Supporting Information Table S1.

### 2.3 RNA-seq and data analysis

Total RNA was isolated from cultured cells or frozen tissues using TRIzol™ reagent (ThermoFisher, 15596026). Library constructions were performed using the NEBnext Ultra ii Stranded RNA Library Prep Kit, and sequencing was performed on an Illumina NextSeq 500 system (50 × 50 bp paired end reads). Data were trimmed using trim-glare and the resultant read sequences were aligned to the hg19 human reference genome using hisat2 aligner. Aligned reads in SAM format were counted using HTSeq and transformed to FPKM in R software. Only protein-coding genes were selected for further analysis. Genes were ranked based on fold changes (FC) and genes with |FC| > 1.5 were subject to Gene Ontology (GO) analysis in R using clusterProfiler package. Representative GO pathways were displayed using ggplot2 function. Gene Set Enrichment Analysis (GSEA v.4.1) was applied to rank genes based on the log_2_FC with default parameters.

### 2.4 Bioinformatic analyses with data from clinical tumors

Beltran datasets for 34 adenocarcinoma and 15 t-NEPC samples were downloaded from cBioPortal website (https://www.cbioportal.org/study/summary?id=nepc_wcm_2016). GSE126078 datasets were downloaded from the Gene Expression Omnibus (GEO) database. The patients were grouped according to the clinical classification and the expression of *BRD4* was analyzed in adenocarcinoma and NEPC subtypes. Signature score of LP programs for each sample in NEPC subtype was generated with R “GSVA” package using gene sets from GSEA database. Based on the signature scores and the gene profile across the samples, the Pearson correlation metric was computed between each gene (*i.e.*, *BRD4*) and each LP signature scores using the “cor” function in GraphPad software.

### 2.5 ChIP-seq and data analysis

BRD4 and H3K27ac ChIP assays were performed with LuCaP145.2 tumor tissues and 42D cells. 2 × 10^7^ 42D cells were transfected with *siFOXA1* or siCtrl for 24 h LuCaP145.2 tumor tissues (approximately 200 mg) were dissected from mice that were treated with either 10 mg/kg of AZD5153 or 50 mg/kg of JQ1 for 7 days before harvest. For tumor tissue, single cells were obtained by homogenizing using cold pellet pestles (Sigma, Z359955) in cold PBS supplemented with protease inhibitor (Roche, 11697498001). Single cells resuspended in PBS were fixed with 1% formaldehyde at room temperature for 8 min, and subsequently quenched with 0.125 mol/L glycine for another 8 min. Cells were washed with cold PBS and suspended with lysis buffer (50 mmol/L HEPES, pH 8.0, 140 mmol/L NaCl, 1 mmol/L EDTA, 10% glycerol, 0.5% NP-40, and 0.25% Triton X-100). Cell pellets were then resuspended in washing buffer (10 mmol/L Tris, pH 8.0, 1 mmol/L EDTA, 0.5 mmol/L EGTA, and 200 mmol/L NaCl), washed, and resuspended in shearing buffer (0.1% SDS, 1 mmol/L EDTA, pH 8, and10 mmol/L Tris-HCl, pH 8). Sonication was performed using Covaris E220 following the manufacturer's instruction. Soluble fractions of sheared chromatin were immunoprecipitated by magnetic protein G beads coated with specific antibodies at 4 °C overnight. Chromatin-captured beads were washed five times with LiCl wash buffer (100 mmol/L Tris, pH 7.5, 500 mmol/L LiCl, 1% NP-40, and 1% sodium deoxycholate), and one time with TE buffer (10 mmol/L Tris, pH 7.5 and 0.1 mmol/L EDTA) before the chromatin fragments were eluted by elution buffer (1% SDS and 100 mmol/L NaHCO_3_). The eluant was incubated with RNase A for 30 min at 37 °C, followed by overnight incubation at 65 °C for reverse crosslinking. After proteinase K treatment, ChIP DNA were purified using PCR purification kit (Qiagen, #28104). Purified ChIP DNA was then used for ChIP-qPCR analysis and library generation. Libraries were prepared, analyzed with the Bioanalyzer 2100 (Agilent) and sequenced in either single-end (BGI, Hong Kong, China) or paired-end (Novogene, USA) 50-bp mode on Illumina Sequencers. ChIP-seq validated antibodies against BRD4 (abcam, #ab ab272042) and H3K27ac (Diagenode, C15410196) were used as indicated in Table S1.

Single-end or paired-end ChIP-seq fastq files were trimmed using Trim Galore v0.6.10 (https://anaconda.org/bioconda/trim-galore) and reads were aligned to the hg19 human genome with Bowtie2 v 2.5.1 (https://anaconda.org/bioconda/bowtie2). PCR duplicates in BAM files were removed with samtools mardup function. Significant ChIP-seq peaks were called using MACS2 (v 2.2.7.1) using a ChIP input file as a control with *P*-value <0.05 in narrow peak mode (for FOXA1) or broad peak mode (for BRD4 and H3K27ac). The heatmaps and binding profiles of ChIP-seq data were generated by deeptools program using default parameters. The shared and unique peaks between various ChIPseq and ATACseq samples were generated using bedtools. The visualization of binding profiles at chromatin were displayed using IGV. For differential binding analysis, normalized binding intensities and log_2_FC for each peak were generated by Manorm algorithm. Because multiple peaks may be annotated with the same gene, the peak with the highest absolute value log_2_FC for each gene was kept for GSEA and GO analyses using the same parameters as RNA-seq analysis. Representative GO and GSEA pathways were displayed using ggplot2 function.

### 2.6 ATAC-seq and data analysis

ATAC-seq experiments were performed using LuCaP145.2 tumor tissues. Fresh tumor tissues were digested into single cell suspension as described in organoids culture and then 50,000 variable cells were used for transposase-accessible chromatin experiments following the procedures as described^33^. ATAC-seq data analysis was performed using nf-core ATAC-seq pipeline with default parameters. Deeptools, bedtools, IGV and Manorm were used for the downstream analysis as described in ChIP-seq analysis.

### 2.7 Establishment of ENZ-resistant PDX tumor models and animal experiments

The compounds enzalutamide, AZD5153 and JQ1were fully dissolved in a formulation of 15% Cremophor EL, 82.5% PBS, and 2.5% DMSO. SCID C.B-17 mice were purchased from Envigo. All mouse experiments were conducted under animal protocols approved by Institutional Animal Care and Use Committee (IACUC) of University of California, Davis. LuCaP35ENZR tumors were derived from LuCaP35CR as follows. Castrated mice carrying LuCaP35CR tumors at approximately 100 mm^3^ were treated with 20 mg/kg ENZ (*p*.*o*.) 5 times per week, for 45 days before the tumor grew to approximately 1000 mm^3^. The tumor tissues were dissected and reimplanted to additional castrated mice and mice were treated as above for another 45 days before the re-grown tumors were harvested and reimplanted for the experiments. To establish xenograft tumor models for the treatments, H660, LuCaP145.2 and LuCaP35ENZR fresh tumors were isolated from mice and propagated by inserting ∼2 mm^3^ into right flank of each mouse. To achieve statistical significance, five to six mice were randomized to different groups when the tumors reached approximately 100 mm^3^. Mice were then treated daily with AZD5153 (10 mg/kg, i.p.), JQ1 (50 mg/kg, i.p.) or vehicle for around 3 weeks depending on the model. For LuCaP35ENZR, all the mice were treated with 20 mg/kg ENZ (*p*.*o*.) to keep the resistant features. Tumor volumes were measured by using calipers with volume calculated using Eq. (1):

(1) $$\text{Tumor}\;\text{volumes}=\left(\text{Length}\times {\text{Width}}^{2}\right)/2$$

### 2.8 Statistical analysis

Cell culture experiments were conducted with three replicates. GraphPad Prism7 was used for computing *P* values and statistical significance. Data were presented as mean ± standard deviation (mean ± SD). Differences between the treatments were made by Student's paired *t* test and *P* < 0.05 was considered statistically significant.

The remaining materials and methods are described in Supporting Information

## 3 Results

### 3.1 Pharmacological probing of key epigenetic regulators identified BRD4 as a crucial growth and survival factor of NEPC cells

**Figure 1:**
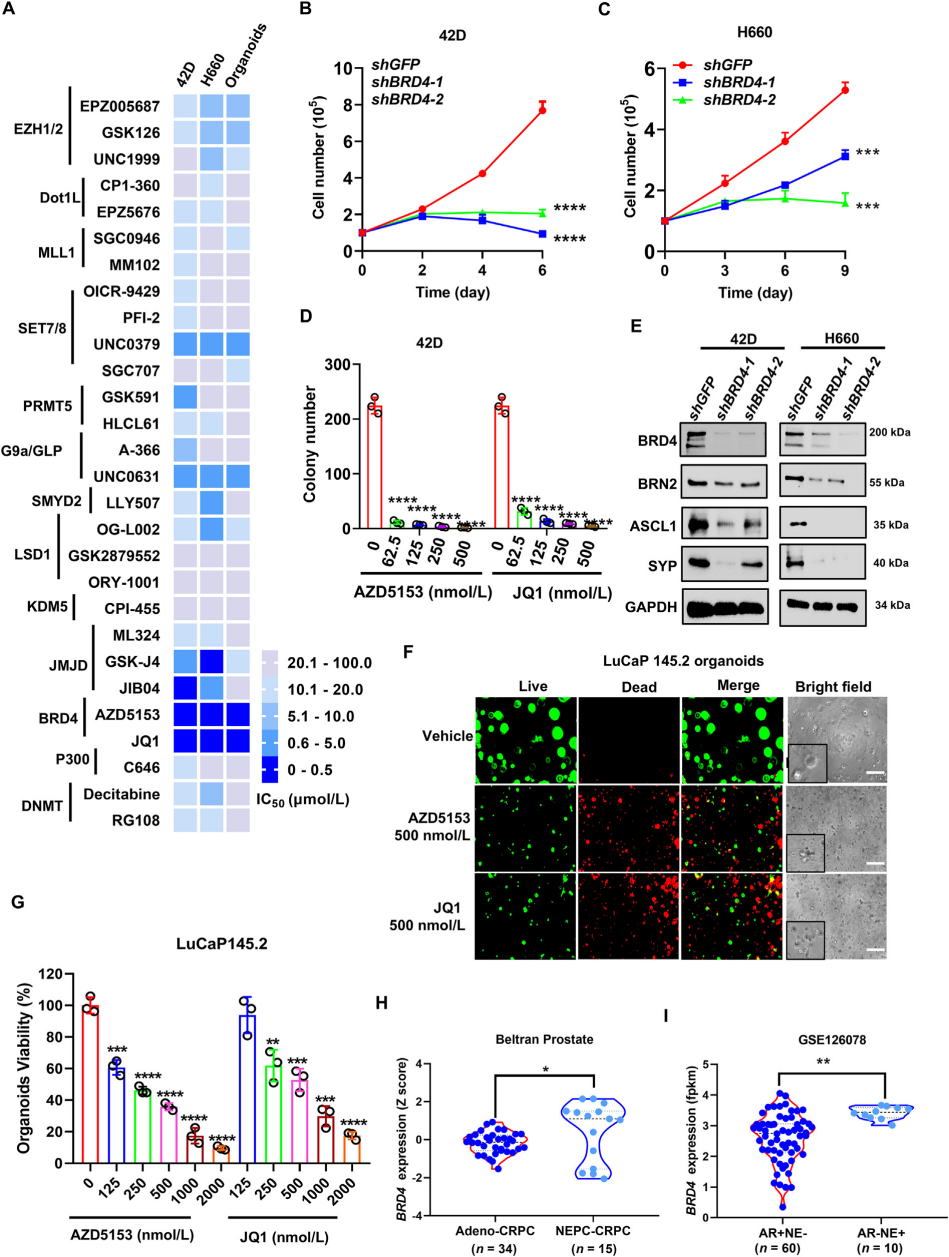
*BRD4* is overexpressed in NEPC tumors and required for NEPC cell growth and survival. (A) Heatmap of IC_50_ in 42D and H660 cells and LuCaP145.2 NEPC PDX tumor derived organoids which were treated with the epigenetic inhibitors for 4 days. Cell viability measured by CellTiter-Glo® 3.0 reagent. (B, C) 42D and H660 cells were infected with lentivirus expressing control shRNA against *GFP* or two different shRNAs against *BRD4* for indicated times. Cell growth curves were plotted. (D) 42D cells were treated with indicated concentrations of BRD4 inhibitors JQ1 and AZD5153 for 14 days. Cell colonies were counted. (E) 42D and H660 cells were infected with lentivirus containing shRNAs targeting *BRD4* for 72 h, before the expression of BRD4 and NEPC drivers BRN2, ASCL1 and NEPC marker SYP was detected by immunoblotting. (F, G) LuCaP145.2 PDX-derived organoids were treated with DMSO or indicated concentrations of BRD4 inhibitors. Four days later, representative images were taken under a fluorescence microscope or standard light microscope (F). Scale bar = 200 μm. Organoid viability was measured using CellTiter-Glo (G). (H, I) The expression of BRD4 in adenocarcinoma subtype and NEPC subtype in Beltran cohort and GSE126078 cohort. Data are shown as mean ± SD. *n* = 3. Student's *t* test. ∗∗*P* < 0.01, ∗∗∗*P* < 0.001, ∗∗∗∗*P* < 0.0001.

Epigenetic reprograming plays vital roles in NEPC progression. However, very few modulators targeting epigenetic regulators were demonstrated to have high efficacy in NEPC. To identify new therapeutic targets for NEPC, we performed a limited scale screening of inhibitors targeting histone or DNA methylases, demethylases and bromodomain proteins. We found that BRD4 inhibitor AZD5153 and JQ1 displayed the most potent inhibition with an IC_50_ lower than 500 nmol/L in t-NEPC 42D cells, *de novo* NEPC H660 cells and a PDX-derived organoids (Fig. 1A). Interestingly, most of the compounds targeting other epigenetic regulators such as EZH2 displayed relatively high IC_50_ values (>5 μmol/L) (Fig. 1A). Notably, AZD5153, a bivalent BRD4 inhibitor, displayed desirable safety and efficacy profile in phase II clinical trials for non-NEPC cancer types^31^. To examine the function of BRD4 in NEPC, we knocked down BRD4 in both 42D and H660 cells and found that BRD4 depletion potently inhibited cell growth and survival (Fig. 1B and C; Supporting Information Fig. S1B). The knockdown also strongly suppressed the expression of NEPC drivers (*POU3F2* and *ASCL1*) and NEPC marker (*SYP*) (Fig. 1E). Consistent with the results from screening, BRD4 inhibitors AZD5153 and JQ1 potently inhibited colony formation of the NEPC cells in a dose-dependent manner (Fig. 1D and Fig. S1A). Treatment of organoids derived from two different NEPC PDX tumors also showed that the two BRD4 inhibitors displayed highly potent inhibitory effects on the viability of the organoids (Fig. 1F and G, Fig. S1C and S1D). These results indicated that BRD4 may play a similar role in t-NEPC and *de novo* NEPC, which is consistent with previous findings that t-NEPC and *de novo* NEPC share similar epigenetic landscapes^15^. In line with the crucial role of BRD4, its expression is significantly higher in both t-NEPC and *de novo* NEPC subtypes, compared to adenocarcinomas (Fig. 1H and I). Moreover, the elevated expression of *BRD4* significantly correlated with poor survival of prostate cancer patients (Fig. S1E). Taken together, our findings suggest that BRD4 plays a crucial role in growth and survival of NEPC cells.

### 3.2 BRD4 controls the induction of lineage plasticity (LP) programs and key NEPC drivers

**Figure 2:**
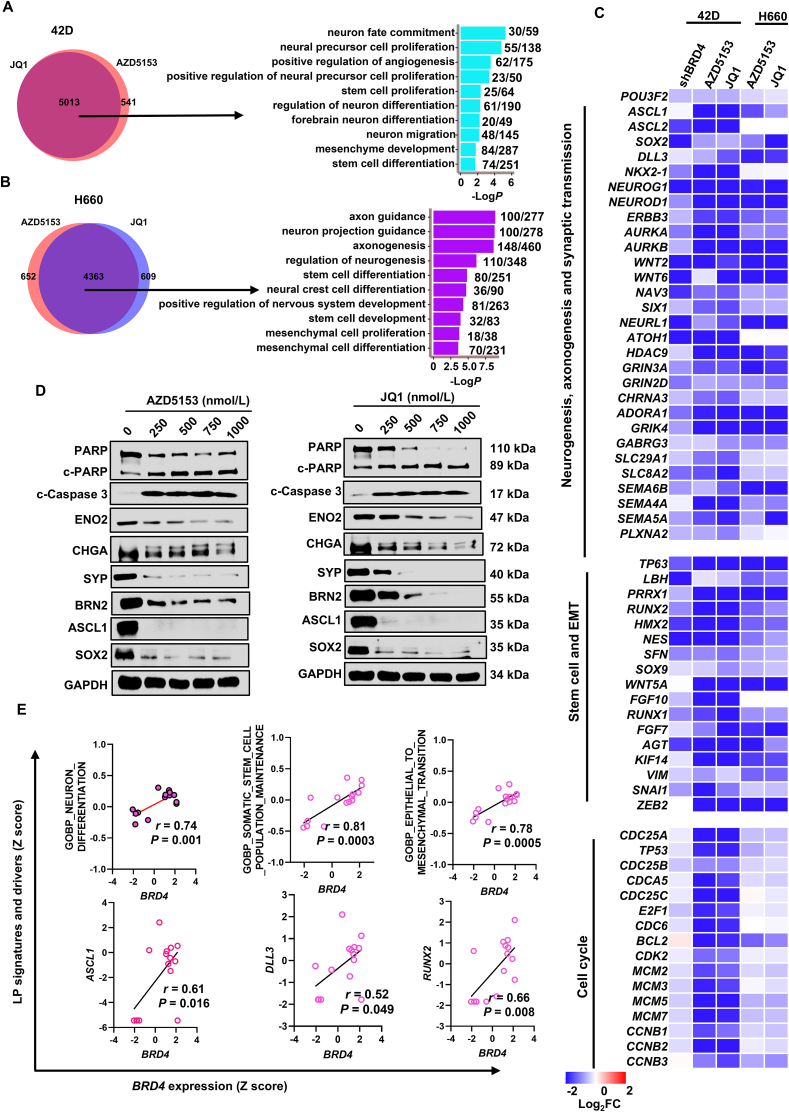
BRD4 controls lineage plasticity (LP) programs in NEPC. (A, B) Left, Venn diagram of the number of protein-coding genes with expression significantly (>1.5-fold) downregulated in t-NEPC 42D and *de novo* NEPC H660 cells treated with 500 nmol/L JQ1 and 500 nmol/L AZD5153 for 48 h, respectively. Right, Gene ontology (GO) analysis of the commonly down-regulated genes by JQ1 and AZD5153 treatment. Top 10 representative programs were shown. Also shown at right are the number of downregulated genes and the total number of genes in each program. (C) Heatmap shows mRNA expression changes of genes in cell cycle and LP programs including neurogenesis, stem cell and EMT identified by GO analysis in 42D and H660 cells. (D) Immunoblotting of proteins involved in neuronal programs and apoptosis in H660 cells treated with indicated concentrations of BRD4 inhibitors AZD5153 and JQ1 for 2 days. The experiments were repeated three times. (E) Correlation between the expression of *BRD4* and LP programs and drivers in NEPC subtype (*n* = 15) in Beltran cohort. ∗∗*P* < 0.01, ∗∗∗*P* < 0.001, ∗∗∗∗*P* < 0.0001.

To understand the functional mechanism of BRD4 in NEPC, we first performed RNA-seq analysis of NEPC cells with *BRD4* knockdown or inhibition by AZD5153 and JQ1. Gene ontology (GO) analysis of the 3167 genes that were downregulated (log_2_FC > 1.5) after *BRD4* knockdown revealed that programs including neurogenesis, stem cell and epithelia mesenchymal transition (EMT), which hereafter are referred to as lineage plasticity (LP) programs, were significantly enriched (Supporting Information Fig. S2A, Tables S3 and S7). Further examination by GSEA also indicated clearly that hallmarks of neuron differentiation, stem cell proliferation and EMT were strongly inhibited by *BRD4* knockdown (Fig. S2B). Interestingly, genes that were downregulated by BRD4 inhibitors AZD5153 and JQ1 were largely overlapping (nearly 90%) in t-NEPC 42D cells and *de novo* NEPC H660 cells, suggesting that those two compounds exerted a highly similar on-target activity in both types of NEPC cells (Fig. 2A and B, left; Supporting Information Tables S4 and S5). Like the effects of BRD4 knockdown, genes downregulated by the inhibitors were also highly enriched in LP programs including neurogenesis, stem cell and EMT, especially in H660 cells where, for example, 148 out of total 460 genes in axonogenesis were downregulated (Fig. 2A and B, right; Supporting Information Tables S8 and S9). Further examination by gene-set enrichment analysis (GSEA) also indicated clearly that hallmarks of neuron differentiation, stem cell proliferation and EMT were strongly inhibited by the BRD4 inhibitor AZD5153 (Figs. S2C and S2D). Several key drivers^13^, ^14^, ^15^^,^^34^, ^35^, ^36^ of LP programs in NEPC progression such as *POU3F2*, *ASCL1/2*, *SOX2/9*, *SNAI1* and *RUNX1/2* were commonly decreased by BRD4 knockdown and pharmacological inhibition (Fig. 2C). Consistently, the BRD4 inhibitors strongly downregulated the protein expression of LP drivers BRN2, ASCL1, SOX2, Snail, Slug and NEPC markers SYP, ENO2 and CHGA and EMT markers vimentin and claudin-1 (Fig. 2D, Fig. S2E). Moreover, we tested the effects of BD1 and BD2 bromodomain-specific inhibitors, iBET-BD1 [GSK778] and iBET-BD2 [GSK046] on the expression of genes in LP programs in t-NEPC 42D cells and found that iBET-BD1[GSK778] showed much less potent effects when compared to AZD5153 and that iBET-BD2 [GSK046] had no effects on the expression of LP genes (Fig. S2F). In support of the notion that BRD4 is a major driver of the LP programs in NEPC tumors, the expression of BRD4 in clinical tumors had a significant, positive correlation with the expression of specific LP programs and drivers such as *ASCL1*, *DLL3* and *RUNX2* (Fig. 2E). In line with the potent effects of BRD4 inhibitors in induction of cell death as shown in Fig. 1F and Fig. S1C, the inhibitors significantly upregulated the level of apoptosis-associated cleavage of PARP1 and caspase3 in a dose-dependent manner (Fig. 2D). Together, these results suggest that BRD4 is a major driver of LP programs in NEPC.

### 3.3 BRD4 inhibitor AZD5153 displayed higher potency than JQ1 in inhibition of NEPC tumor growth and LP programs *in vivo*

**Figure 3:**
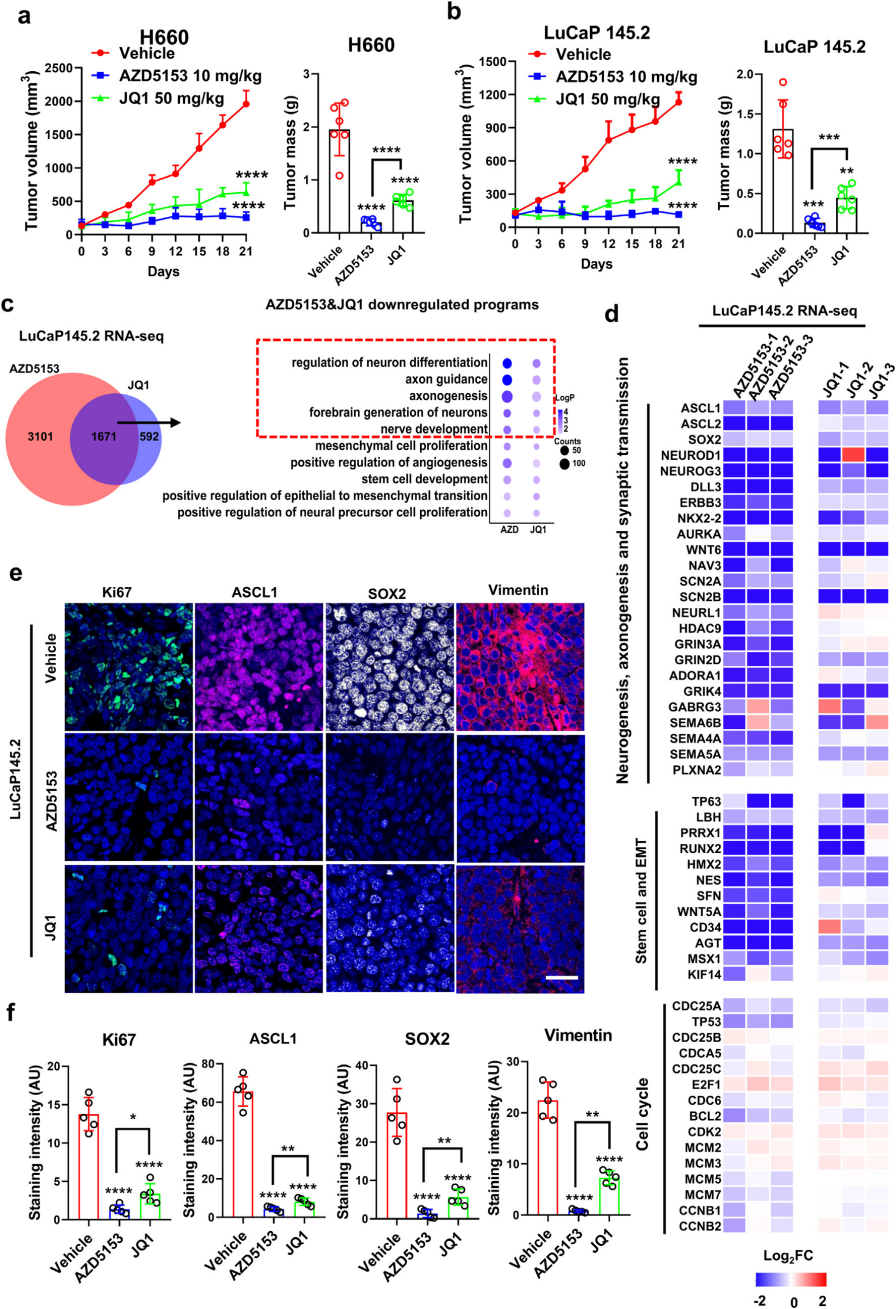
BRD4 inhibitors potently inhibited NEPC tumor growth and LP programs *in vivo*. (A, B) Mice bearing H660 and LuCaP145.2 tumors (*n* = 6) were treated, i.p., 5 times per week, with vehicle, 50 mg/kg JQ1 and 10 mg/kg AZD5153 for 21 days. Tumor volume was measured every 3 days and tumor growth curves were drawn to show growth of tumors of each group. (C–F) When tumor reached around 300 mm^3^, mice bearing LuCaP145.2 tumors were treated i.p. with vehicle, 50 mg/kg JQ1 and 10 mg/kg AZD5153 for 7 consecutive days and tumor tissues were preserved in OCT for immunofluorescence or snap frozen for protein and RNA analysis. (C) Venn diagram of the number of protein-coding genes with expression significantly (1.5-fold) downregulated, which is detected by RNA-seq of PDX tumors treated with JQ1 and AZD5153, respectively. Gene ontology (GO) analysis of genes downregulated by JQ1 and AZD5153 treatment. Top 10 representative programs are shown. (D) Heatmap display of relative expression changes (compared to Vehicle) of genes in LP programs including neurogenesis, axonogensis, synaptic transmission, stem cell and EMT and in cell cycle programs. (E, F) Ki67, ASCL1, SOX2 and Vimentin immunofluorescence were performed. Representative images from three independent tumors are shown. Staining intensity of these proteins were measured by image J using at least 5 random images. Data are shown as mean ± SD. Student's *t* test. ∗∗*P* < 0.01, ∗∗∗*P* < 0.001, ∗∗∗∗*P* < 0.0001.

Given our results that BRD4 controls NEPC drivers and crucial for NEPC cell growth and survival, we next evaluated the therapeutic potential of BRD4 inhibitors AZD5153 and JQ1 using several enzalutamide (ENZ)-resistant NEPC models. First, in two *de novo* NEPC models (H660 xenografts and LuCaP145.2 PDX), intraperitoneal administration of AZD5153 at a relatively low dose (10 mg/kg) was sufficient to achieve almost complete blockade of the tumor growth, without discernible effects on the animal body weight (Fig. 3A and B, Supporting Information Fig. S3A–S3D). However, treatment with JQ1, at 50 mg/kg, did not result in a complete blockade, although a strong inhibition of the tumor growth was observed (Fig. 3A and B). We developed an AR-positive, ENZ-resistant PDX subline from a CRPC model LuCaP35CR through treatment of the tumor-carrying mice with 20 mg/kg ENZ (*p*.*o*.) (see Supporting Information methods for details)^37^. Treatment of mice carrying the LuCa35ENZR tumors with AZD5153 elicited a potent inhibitory effect with the tumor growth being completely blocked over the course of treatment (Fig. S3E).

To understand the mechanism underlying the potent anti-tumor effects of BRD4 inhibitors, we performed RNA-seq analysis of treated tumors. Consistent with the effects observed in cell culture, both AZD5153 and JQ1 downregulated expression of genes enriched in the LP programs. Interestingly, AZD5153 displayed much higher potency (in the number of genes and –Log*P* values) than JQ1 in inhibition of the LP programs including regulation of neuron differentiation, axonogenesis and nerve development (Fig. 3C, Supporting Information Tables S6, S10 and S11). Indeed, the expression of key drivers and factors in neurogenesis (*ASCL1*, *NeuroD1*, *NKX2-2*), stem cell and EMT (*SOX2/9*, *RUNX1/2*, *ZEB2*, *VIM* and *NNMT*) was strongly inhibited by AZD5153 in almost all the tumors examined (Fig. 3D). However, their expression was only moderately affected by JQ1 (Fig. 3D). GSEA analysis also showed that signature genes of nervous system development, stem cell and EMT were more significantly reduced by AZD5153 than JQ1 (Fig. S3H). In contrast, regulation of protein secretion was more significantly affected by JQ1 than AZD5153 (Fig. S3H). Our further analysis of protein expression in tumors by immunofluorescence (IF) also revealed that AZD5153 displayed higher potency than JQ1 in suppression of NEPC drivers ASCL1 and SOX2, which were homogenously expressed in the *de novo* NEPC tumors and heterogeneously expressed in the ENZR tumors (Fig. 3E and F, Fig. S3F and S3G). Together, our findings from these animal experiments strongly suggest that BRD4 inhibitors, particularly AZD5153, are highly effective in blocking growth of NEPC and ENZ-resistant tumors, through inhibition of expression of key NEPC drivers and LP programs. Moreover, our results demonstrated that AZD5153 possesses a higher potency than JQ1 in tumor growth inhibition and suppression of the NEPC gene expression.

### 3.4 BRD4 is reprogrammed by ARPIs and displays highly similar cistromes in t-NEPC and *de novo* NEPC

**Figure 4:**
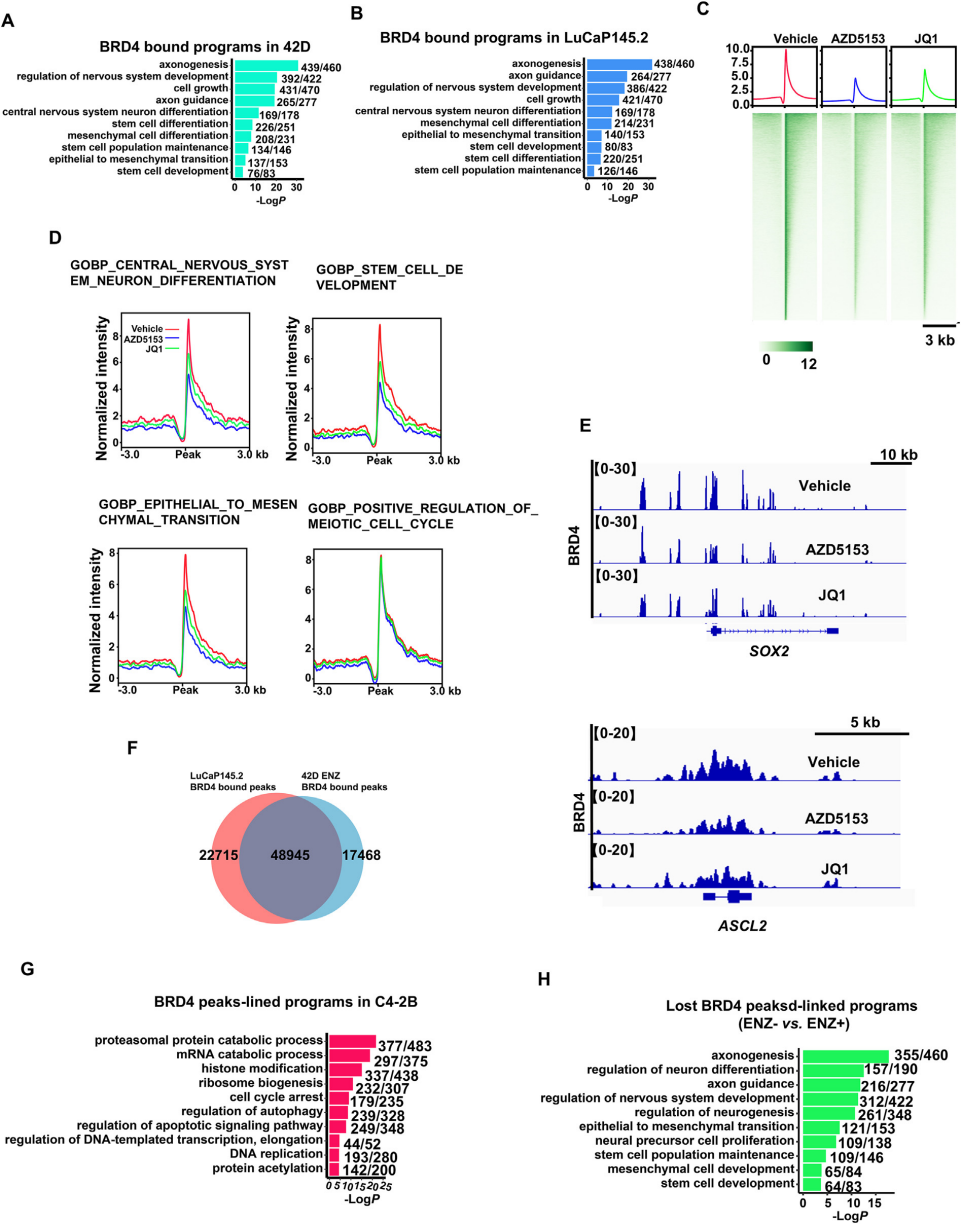
BRD4 is reprogrammed by ENZ and directly controls LP programs at both promoters and enhancers in NEPC. (A) GO analysis of BRD4 ChIP-seq peaks-linked genes in 42D cells. Top 10 representative LP programs are shown. Also shown are the number of genes with BRD4 ChIP-seq peaks and the total gene number of each program. (B) GO analysis of BRD4 ChIP-seq peaks-linked genes in LuCaP145.2. Top 10 representative LP programs are shown. Also shown are the number of genes with BRD4 ChIP-seq peaks and the total gene number of each program. (C–E) LuCap145.2 tumors were treated with AZD5153 (10 mg/kg, i.p.) or JQ1 (50 mg/kg, i.p.) for 7 days and then tumor was harvested for BRD4 and H3K27ac ChIP-seq analysis. (C) Heatmap representation of ChIP-seq signal of BRD4 at peak center. (D) BRD4 ChIP-seq peak profile within 3-kb windows around the center of BRD4 peaks at genes in LP and cell cycle programs in LuCaP145.2 tumors treated with BRD4 inhibitors. (E) IGV snapshots of BRD4 occupancy at *SOX2* and *ASCL1* chromatin region at promoters and enhancers. (F) Venn diagrams show the overlap of BRD4 peaks in t-NEPC 42D cells and *de novo* NEPC LuCaP145.2 tumors. (G) GO analysis of BRD4 peaks-linked genes in C4-2B cells. Top 10 representative LP programs are shown. (H) Differential binding analysis of BRD4 in 42D cells with or without ENZ treatment. The peaks lost without ENZ treatment were subjected to GO analysis and top 10 representative LP programs were shown. Also shown are the number of genes with BRD4 ChIP-seq peaks and the total gene number of each program.

The high potency of the BRD4 inhibitors in blocking different NEPC models prompted us to further study the function and mechanism of BRD4. Thus, we performed ChIP-seq analysis of BRD4 cistromes in t-NEPC and *de novo* NEPC cells and tumors. We found that among 66,415 peaks in t-NEPC cells and 71,660 peaks in *de novo* NEPC tumors, over 65 percent of the BRD4 ChIP-seq peaks were mapped to the enhancers (Supporting Information Fig. S4A; Tables S17 and S18). Analysis of peaks-linked genes revealed that, in both t-NEPC and *de novo* NEPC, BRD4 binds to the vast majority (range from 85% to 95%) of genes in the LP programs (Fig. 4A and B; Supporting Information Tables S19 and S20). In t-NEPC 42D cells, BRD4 binds to 439 genes in axonogensis program (*e*.*g*., *POU3F2*/BRN2 and *NKX2-1*), 329 genes in regulation of nervous system development (*e*.*g*., *SOX2*, *ASCL1* and *ASCL2*), 137 genes in EMT and 76 genes in stem cell development (*e*.*g*., *SOX9* and *VIM*) (Fig. 4A and B, Fig. S4B and S4C). In *de novo* NEPC tumors, BRD4 binds to similarly high number of genes in the four programs (Fig. 4A and B, Fig. S4B). In line with a previous study^27^, BRD4 also binds to genes that are controlled by E2F1, such as genes involved in control of cell cycle and genes in regulation of apoptotic signaling pathway (Fig. S4B). Strikingly, treatment of BRD4 inhibitors, particularly AZD5153, strongly inhibited BRD4 binding at the LP programs (Fig. 4C–E). In contrast, the inhibitors did not have significant effects on the E2F1-controlled gene programs such as meiotic cell cycle (Fig. 4D and Fig. S4F).

The high concordance of BRD4 binding-linked gene programs in t-NEPC and *de novo* NEPC suggests that BRD4 may control the LP programs through binding to similar genomic locations. Indeed, approximately 80% of BRD4 peaks are overlapping in the two systems and they are linked to the same LP programs, suggesting that BRD4 binds to the same regulatory regions to control the LP programs in t-NEPC and *de novo* NEPC (Fig. 4F). To understand whether BRD4 cistrome at LP programs is preexisted in the ARPIs-sensitive cells or reprogramed in the progression, we analyzed BRD4 cistromes in the CRPC cells (C4-2B) which is isogenic to the t-NEPC cells (42D). Results in Fig. 4G demonstrated that in the CRPC cells, the BRD4 cistrome is enriched in cell survival and cell cycle programs with little to no binding at the LP programs, thus indicating that BRD4 occupancy at LP programs is a function induced in progression to NEPC (Supporting Information Tables S21 and S22). To examine whether ARPI ENZ is a key factor that reprograms BRD4, we analyzed BRD4 cistrome in t-NEPC cells in the presence or absence of ENZ (Fig. 4H). Indeed, upon ENZ withdraw, BRD4 binding at LP programs are significantly reduced (Fig. S4D and S4E). Taken together, our analyses revealed that in t-NEPC and *de novo* NEPC, BRD4 binds to similar genomic sites to predominantly activate the LP programs including key drivers and markers and that such BRD4 function is reprogrammed in the progression to NEPC with ARPIs being a major driving factor.

### 3.5 BRD4 stimulates LP programs through increasing chromatin accessibility

**Figure 5:**
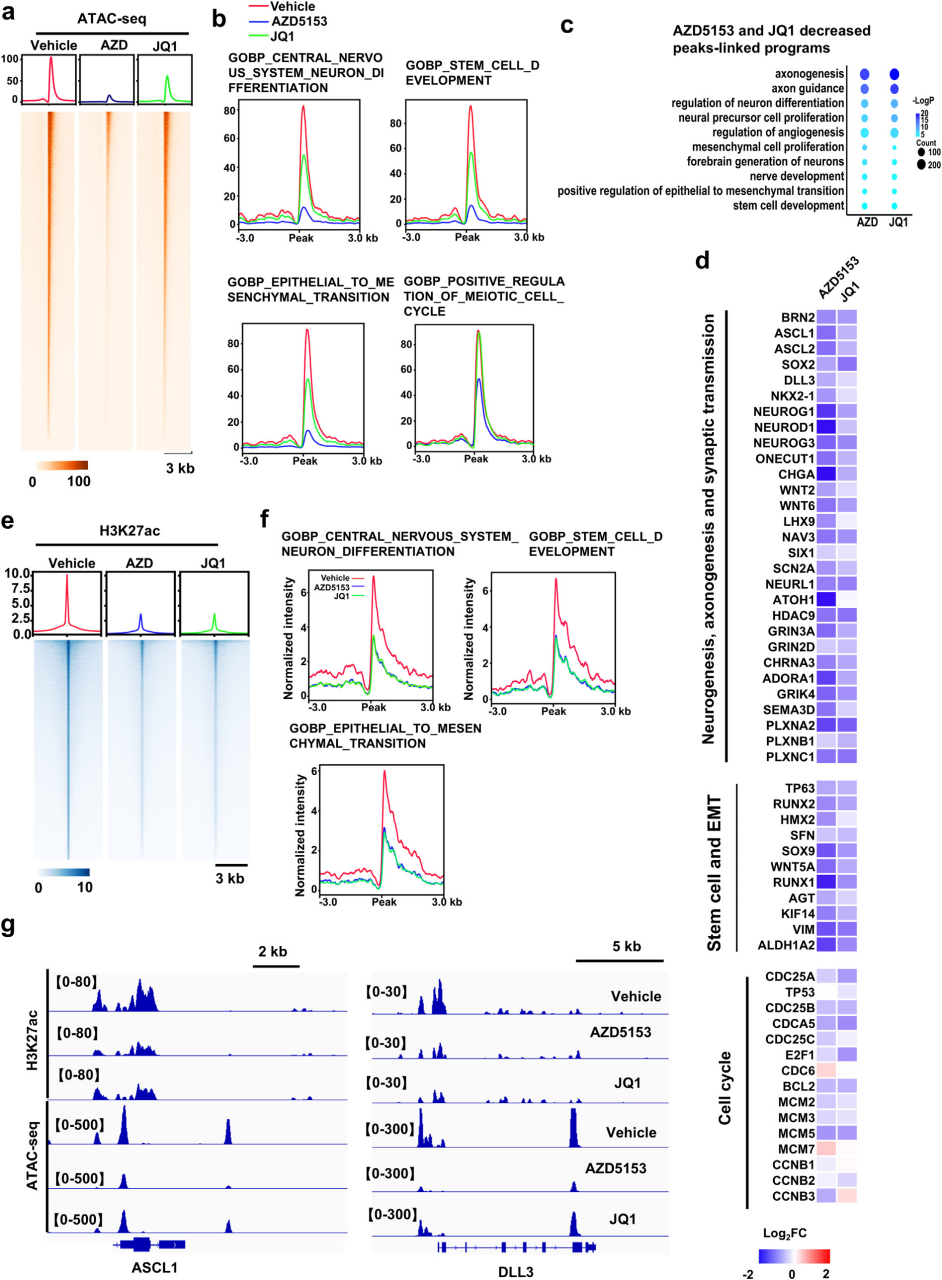
BRD4 stimulates LP programs through increasing chromatin accessibility. (A) Heatmap presentation of genome-wide chromatin accessibility detected by ATAC-seq in LuCaP145.2 tumors. (B) Chromatin accessibility profile within ±3-kb windows around peak center at LP and cell cycle programs. (C) Bubble plots show enrichment of LP-associated, GO gene programs with ATAC-seq peaks decreased by AZD5153 and JQ1. (D) Heatmaps display the log_2_ fold change of chromatin accessibility at genes in LP programs including neurogenesis, stem cell and EMT. (E) Heatmap presentation of ChIP-seq signal of H3K27ac at peak center. (F) H3K27ac signal within ±3-kb windows around the center of H3K27ac peaks at genes in LP programs including neurogenesis, stem cell and EMT in LuCaP145.2 tumors treated with BRD4 inhibitors. (G) IGV snapshots of chromatin accessibility and H3K27ac signal at locus of LP drivers *ASCL1* and *DLL3*.

BRD4 has been demonstrated to possess chromatin remodeling activities^23^. To better understand the predominant role of BRD4 in controlling the LP programs, we performed ATAC-seq of tumors treated by BRD4 inhibitors. Strikingly, the two inhibitors dramatically decreased local chromatin accessibility (Fig. 5A and B). Unbiased analysis of chromatin regions with reduced ATAC-seq peaks demonstrated that the inhibitors preferentially reduced the chromatin accessibility at LP programs, such as neuron differentiation, stem cell and EMT (Fig. 5C and Supporting Information Fig. S5A, Tables S12–S16). Consistent with the lack of effects on the BRD4 binding at the cell cycle programs, the treatment did not significantly affect the local chromatin accessibility (Fig. 5B). Quantitative analysis of the peaks revealed that AZD5153 displayed a higher potency than JQ1 in decreasing the local chromatin accessibility at LP programs including LP drivers *ASCL1*, *RUNX2* and *SOX2* (Fig. 5C and D; Fig. S5B and S5C).

One function of BRD4 at chromatin is to maintain local H3K27ac acetylation by recruiting acetylase such as CBP/p300^38^^,^^39^. Also, a previous study demonstrated that BRD4 can display a histone acetyltransferase (HAT) activity to acetylate H3 and H4^23^. As expected, the two BRD4 inhibitors strongly decreased H3K27ac mark genome wide and at LP programs (Fig. 5E and F). Intriguingly, AZD5153 and JQ1 displayed almost equivalent potency in the reduction of H3K27ac (Fig. 5E–G). Taken together, our results suggest that the strong effects of disruption of BRD4 binding and decreasing local chromatin accessibility constitute the major mechanism of action of AZD5153 in suppression of the LP programs *in vivo*. These results also suggest that BRD4 stimulates LP programs through increasing local chromatin accessibility.

### 3.6 FOXA1 facilitates BRD4 recruitment to the LP gene targets

**Figure 6:**
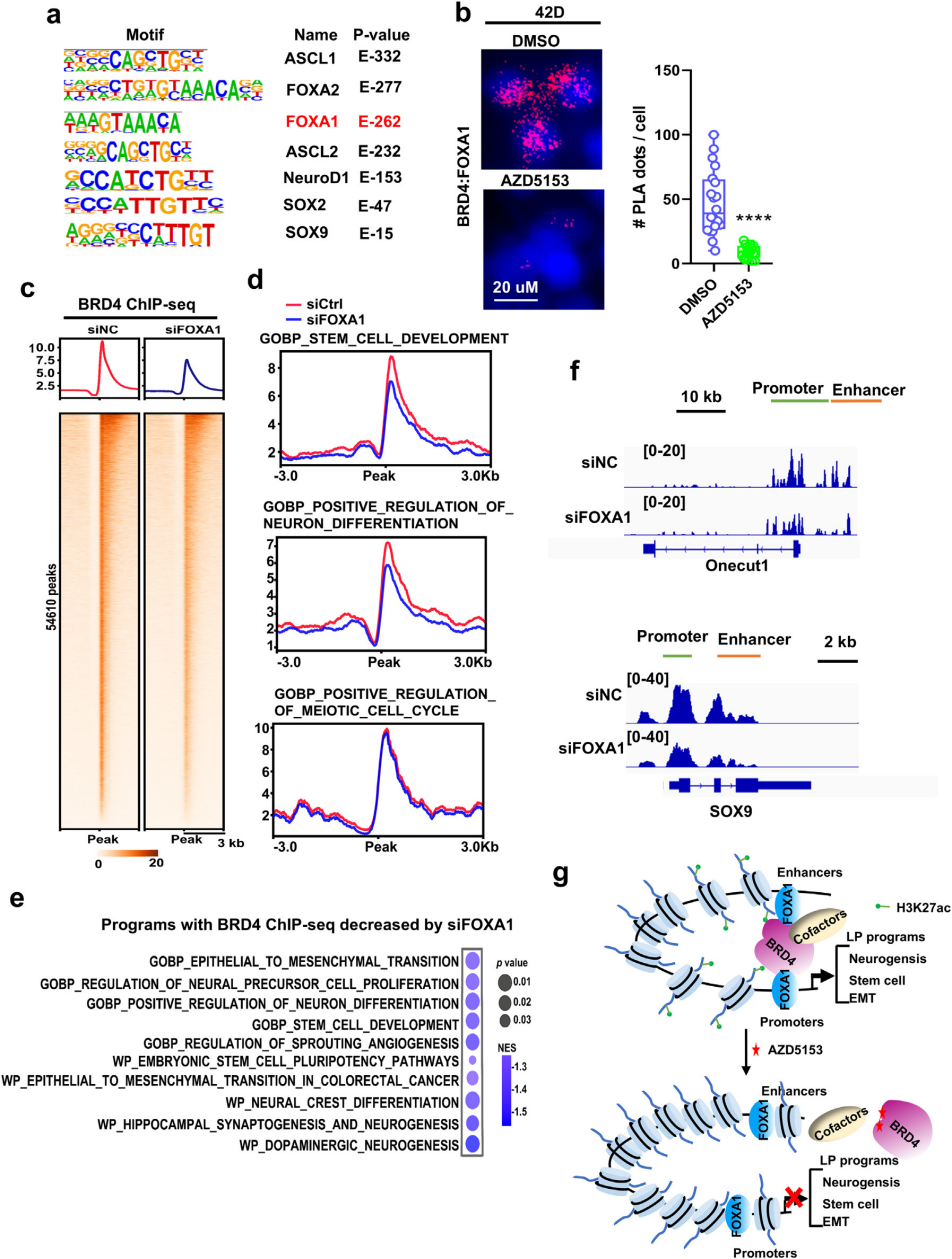
Chromatin binding of BRD4 is promoted by reprogrammed FOXA1 in NEPC. (A) Motif enrichment analysis of ATAC-seq differential peaks, listing 7 representative TFs linked to LP. (B) Proximity Ligation Assay (PLA) for protein interactions between BRD4 and FOXA1 in 42D cells. The cells were treated with 500 nmol/L AZD5153 for 24 h, and then Duolink assay between BRD4 and FOXA1 was performed (cells without treatment as positive control). (C) Heatmap presentation of ChIP-seq signal of BRD4 after *FOXA1* knockdown for 24 h at peak center in 42D cells. (D) BRD4-bond profile within ±3-kb windows around the center of BRD4 peak regions at genes of LP and cell cycle programs in 42D cells after *FOXA1* knockdown. (E) Bubble plots show the decreased chromatin occupancy of BRD4 affected by *FOXA1* knockdown at programs involved in LP significantly enriched by GSEA analysis of in 42D cells. (F) IGV snapshots of BRD4 occupancy at chromatin region of LP drivers *NR2F2*, *SOX9*, *ONECUT1* and *ASCL2*. (G) Schematics of a potential mechanism of BRD4 function in NEPC and the effect of the BRD4 inhibitor on NEPC tumors.

Finally, to understand how BRD4 is reprogramed to the LP programs in NEPC, we first performed HOMER motif analysis of the ATAC-seq peaks and ChIP-seq peaks that were decreased by AZD5153. Our analysis identified FOXA1 motif being one of the most significantly enriched (Fig. 6A and Supporting Information Fig. S6A, Tables S27 and S28). Previous studies have shown that FOXA1 is also reprogrammed in NEPC and plays a vital role in NEPC progression^40^. In both co-IP and PLA analysis, we observed that BRD4 strongly interacted with FOXA1 (Fig. S6B and Fig. 6B). The interaction was dramatically inhibited by the BRD4 inhibitors (Fig. 6B). Analysis of FOXA1 cistrome in the NEPC LuCaP145.2 tumors revealed that BRD4 peaks were significantly overlapped with FOXA1 (23,120 peaks, around 30%) (Fig. S6C, left; Table S24). Interestingly, the overlapped peaks were primarily linked to the LP programs (Fig. S6C and S6D, right; Table S23). To examine whether FOXA1 plays a role in reprograming of BRD4, we performed *FOXA1* knockdown followed by BRD4 ChIP-seq. As showing in Fig. 6C–F, knockdown of *FOXA1* strongly inhibited BRD4 bindings, especially at the LP programs. Little to no effects were observed at the other programs including cell cycle (Fig. 6D, right; Tables S25 and S26). Consistent with a reprograming role, *FOXA1* knockdown significantly inhibited expression of the LP program genes and the growth and survival of NEPC cells (Fig. S6E–S6H). Double knockdown of *FOXA1* and *BRD4* resulted in stronger downregulation of the LP drivers and markers including BRN2, ASCL1, SOX2 and SYP (Fig. S6I), suggesting that BRD4 and FOXA1 function cooperatively in induction of LP. Taken together, these results suggest that BRD4 chromatin binding in NEPC tumors is reprogramed by FOXA1.

## 4 Discussion

The function of BRD4 in CRPC prostate cancer is relatively well understood^6^^,^^26^^,^^41^ which has led to clinical trials of BRD4 inhibitors in treating CRPC patients. However, little is known about the role of BRD4 in NEPC. In our search for new therapeutic targets for NEPC, we identified BRD4 as a major player. BRD4 is crucial for growth and survival of multiple NEPC models including PDXs. Our functional genomics approaches revealed that BRD4 directly activates the expression of NEPC drivers and the LP programs. We found that BRD4 overexpression is positively associated with gene signatures of LP programs in the lethal form of PCa. Interestingly, we also found that BRD4 cistrome is reprogramed in progression from CRPC to NEPC which is facilitated by pioneer factor FOXA1. More importantly, our study demonstrated that BRD4 inhibitor AZD5153 holds great promise in effectively treating the lethal form of the disease.

Previously, a study strongly implicated the function of BRD4 in control of specific genes/programs in t-NEPC cells^27^. They demonstrated that enzalutamide treatment of 42D cells resulted in an increased chromatin accessibility at gene programs, such as neurogenesis and neuron differentiation. They also performed ChIP-seq and showed that BRD4 directly binds to specific genes including *BCL2*, *MET*, *KIAA0319* and *CHAC1*. Among those four genes, *KIAA0319* and *CHAC1* may play an important role specifically in neurogenesis. Their further study demonstrated that cell cycle regulator E2F1 is a potential mediator of the BRD4 function^27^. In our study of BRD4 cistromes in NEPC, we found that BRD4 target genes are enriched primarily in the major programs that define LP in NEPC, namely neurogenesis, axonogenesis, stem cell and EMT. For instance, 316 out of 348 genes in regulation of neurogenesis are directly controlled by BRD4, including *NGF*, *BDNF* and *CAMK2B*. 438 out of 460 genes in axonogenesis are directly controlled by BRD4, including *SEMA6B*, *SEMA4A* and *PLXNA2*. Significantly, the major LP drivers such as *POU3F2* (BRN2), *ASCL1/2*, *SOX2*, *RUNX1/2* and *SOX9* are all directly activated by BRD4. Notably, most of those targets are commonly identified in both t-NEPC and *de novo* NEPC cell and PDX models. Their expression is strongly inhibited by BRD4 inhibitors. Therefore, our findings provided, for the first time, the strong evidence that BRD4 plays a fundamental role in NEPC cells and tumors by directly stimulating the expression of key NEPC drivers and hundreds of genes in the LP programs.

Our findings that BRD4 in NEPC primarily controls the LP drivers and gene programs are in stark contrast to the fact that in CRPC adenocarcinomas BRD4 controls primarily AR signaling. Indeed, we found that BRD4 function is reprogramed upon treatment with ARPIs such as ENZ in NEPC. Therefore, loss of AR signaling may be a key mechanism of BRD4 reprograming. Consistent with this notion, our study also revealed that FOXA1, a pioneer factor of AR, facilitates BRD4 binding to the LP programs. Interestingly, FOXA1 itself is reprogrammed to NE-specific regulatory elements in NEPC^40^^,^^42^. Our study here revealed that the cistromes of BRD4 and FOXA1 are largely overlapped at genes in LP programs. The occupancies of BRD4 were significantly diminished upon *FOXA1* knockdown, indicating that FOXA1 is a factor facilitating BRD4 reprograming in NEPC (Fig. 6G). Given the extensiveness of BRD4 reprograming, it is likely that other factors are also involved. For instance, loss of tumor suppressor Rb protein was found to promote BRD4 binding to its specific targets in GPCR–GNBIL–CREB pathway in CRPC models^43^.

One intriguing finding of this study is that BRD4 inhibitor AZD5153 displayed much higher potency than JQ1 in inhibition of NEPC tumor growth. This difference can be partly explained by the different activities of these two inhibitors. AZD5153 displayed a stronger activity than JQ1 in disrupting BRD4 binding and in decreasing local chromatin accessibility (Fig. 5A–D). Interestingly, the two inhibitors displayed similar activity in decreasing H3K27ac mark *in vivo*, suggesting that the stronger antitumor effects of AZD5153 cannot be attributed simply to a possible difference in the drug metabolism or PK profile. One major distinction between these two compounds is that AZD5153 is a bivalent inhibitor that can disrupt binding to acetylated lysines by both BD1 and BD2, whereas JQ1 can disrupt primarily BD1 binding. As reported in a previous study^43^, Rb1 can bind to BD1 of BRD4 and thus inhibit BRD4 function by disrupting BD1 association with acetylated nucleosomes. Most NEPC tumors have lost their Rb expression and/or function^44^^,^^45^. Thus, it is tempting to speculate that the lack of Rb function may decrease the effectiveness of JQ1 in NEPC tumors and that bivalent inhibitors such as AZD5153 could circumvent this problem through disrupting the function of both BD1 and BD2.

## 5 Conclusions

In summary, our study defined the function of BRD4 in NEPC tumors including its crucial role in directly stimulating LP drivers and gene programs. Our study also provided a strong rationale for nominating AZD5153 as a drug candidate in effective treatment of t-NEPC and *de novo* NEPC.

## Data availability

Raw data of RNA-seq, ChIP-seq and ATAC-seq have been publicly deposited in Gene Expression Omnibus database under accession number GSE265762, GSE265759 and GSE265757. Source Data for this study are provided with this paper. All relevant data in this study are available within the article, Supporting Information, or Source Data. Source data are provided with this paper.

## Author contributions

Xiong Zhang: Writing – review & editing, Writing – original draft, Visualization, Validation, Supervision, Software, Resources, Project administration, Methodology, Formal analysis, Data curation, Conceptualization. Yatian Yang: Visualization, Software, Methodology, Formal analysis, Data curation. Hongye Zou: Resources. Yang Yang: Resources. Xingling Zheng: Resources. Eva Corey: Resources. Amina Zoubeidi: Resources. Nicolas Mitsiades: Resources. Ai-Ming Yu: Resources. Yuanpei Li: Resources. Hong-Wu Chen: Writing – review & editing, Writing – original draft, Visualization, Validation, Supervision, Software, Resources, Project administration, Methodology, Investigation, Funding acquisition, Formal analysis, Data curation, Conceptualization.

## Conflicts of interest

The authors declare no competing interests.

## Appendix A Supporting information

The following are the Supporting information to this article:
