# Supplementary material for: Effective therapeutic targeting of tumor lineage plasticity in neuroendocrine prostate cancer by BRD4 inhibitors
Source: Acta Pharm Sin B. 2025 Jan 22;15(3):1415–29. doi: 10.1016/j.apsb.2025.01.007 (PMC12069891; doi:10.1016/j.apsb.2025.01.007)
Supplement: Multimedia component 1 [file mmc1.pdf]

Supporting Information for

Original article

Effective therapeutic targeting of tumor lineage plasticity in neuroendocrine prostate cancer by BRD4 inhibitors

Xiong Zhang[a,†], Yatian Yang[a,†], Hongye Zou[a], Yang Yang[a], Xingling Zheng[a], Eva Corey[b], Amina Zoubeidi[c], Nicolas Mitsiades[d], Ai-Ming Yu[a], Yuanpei Li[a], Hong-Wu Chen[a,e,f,*]

[a]*Department of Biochemistry and Molecular Medicine, School of Medicine, University of California Davis, Sacramento, California 95817, USA*

[b]*Department of Urology, University of Washington, Seattle, Washington 98915, USA*

[c]*Department of Urologic Sciences, University of British Columbia, Vancouver, BC V5Z1M9, Canada*

[d]*Department of Internal Medicine, Division of Hematology and Oncology, School of Medicine, University of California Davis, Sacramento, California 95817, USA*

[e]*Comprehensive Cancer Center, University of California Davis, Sacramento, California 95817, USA*

[f]*VA Northern California Health Care System-Mather, Mather, California 95655, USA*

Received 2 August 2024; received in revised form 13 October 2024; accepted 18 November 2024

*Corresponding author.

E-mail address: hwzchen@ucdavis.edu (Hong-Wu Chen).

[†]These authors made equal contributions to this work.

This file includes:

Materials and methods

Supporting Figures 1–6

Supporting Tables S1–S2

Supporting Tables S3-S28

Materials and Methods

Cell culture and siRNA transfection

All cells were grown in a humidified incubator with 5% CO_2 at 37 °C. NCI-H660 (ATCC, # CRL-5813™) cell line was acquired from ATCC and cultured in HITES medium (RPMI1640 medium plus 0.005 mg/mL insulin, 0.01 mg/mL Transferrin, 30 nmol/L sodium selenite, 10 nmol/L hydrocortisone, 10 nmol/L beta-estradiol and 2 mmol/L L-glutamine) with 5% FBS (Corning, #35-015-CV). Enzalutamide (ENZ)-resistant 42D cells were obtained from Dr. Amina Zoubeidi (University of British Columbia, Canada), cultured in RPMI-1640 containing 10% FBS supplemented with 10 µmol/L ENZ (MCE, # HY-70002) for all experiments unless otherwise stated. All the cell lines used in this research were authenticated by STR profiling and regularly tested being negative for mycoplasma. siRNAs were synthesized by Dharmacon (Cambridge, UK). The siRNA sequences targeting *FOXA1* were 5′-GGACUUCAAGGCAUACGAA-3′ for *siFOXA1-1*, and 5′-GUGUAGACAUCCUCCGUAU-3′ for *siFOXA1-2*. siRNA sequence CAGTCGCGTTTGCGACTGG was used as non-targeting control (siCtrl). Transfection of siRNAs was performed with OptiMEM (Invitrogen, #11058021) and Dharmafect1 (Dharmacon, #T-2001-02), following the manufacturer's instructions.

Chemicals

AZD5153 (S8344) and JQ1 (S6993) (purity > 99.9%) were purchased from Selleck. Other chemicals (purity > 98%) are from MCE and Selleck unless indicated otherwise.

Lentivirus preparation and infection

Two lentiviral shRNAs targeting *BRD4* (*shBRD4-1*, TRCN0000199427; *shBRD4-2* TRCN0000021427) were purchased from Sigma. Lentiviral shRNA targeting *FOXA1* was obtained from Addgene (Plasmid #70095). Lentiviral particles were produced in 293T cells with co-transfection of the shRNA vectors, psPAX2 and pMD2.G in 10-cm dishes. H660 and 42D cells were infected with virus-containing supernatant in the presence of 10 ng/mL polybrene (Sigma, TR-1003-G) for 6 h and maintained in fresh completed medium as indicated before harvested for cell proliferation, protein expression and RNA-seq analysis.

Cell growth and viability assay

For cell growth assay, prostate cancer cells were seeded into 6-well plates at a density of 2×10^5 cells per well. Twenty-four hours later, cells were transfected with siRNA, infected with lenti-virus or treated with compounds as indicated. Live cells were counted using Countess™ II Automated Cell Counter (ThermoFisher) in the presence of trypan blue reagent. For cell viability assay, prostate cancer cells were seeded in 96-well plates at 2000 cells per well in a total volume of 100 μL medium. Twenty-four hours later, cells were treated as indicated. After 4 days of incubation, the medium was discarded and 50 μL Cell-Titer Glo reagents (Promega, G9243) were added and incubated for 5 min. Luminescence was measured by GLOMAX microplate luminometer (Promega), according to the manufacturer's instructions. The above assays were performed in triplicates.

qRT-PCR

Total RNA was isolated from cells in 6-well plates with TRIzol reagent (Invitrogen). One μg of total RNA was reverse transcribed to cDNA using qScript cDNA SuperMix (Quantabio). Then qRT-PCR was performed. Briefly, cDNAs were mixed with SYBR Green master mix (Bimake) and gene specific primers. The PCR was run on a CFX96 connect Real-Time PCR system (Bio-Rad). GAPDH gene transcript was used for normalization. The $2^{-\Delta\Delta CT}$ method was used to obtain the relative quantifications. Experiments were repeated three times. The primers are listed in Supporting Information Table S2.

Immunoblotting and immunoprecipitation

Cell or tissue lysates were analyzed by immunoblotting with antibodies specifically recognizing indicated proteins. The antibodies and dilution ratio used are shown in Supporting Information Table S1. The source data of the uncropped immunoblots are provided in the Source Data file.

For Co-immunoprecipitation (Co-IP) experiments, 42D cell were co-transfected with plasmid expressing Flag-FOXA1 (Addgene, #153109) and HA-BRD4 (Addgene, #31351) in 10 cm dishes overnight and incubated in fresh medium for another 24 h before harvest. Cells were lysed with 400 μL lysis buffer (10 mmol/L HEPES, pH 7.9, 10 mmol/L KCl, 0.1 mmol/L EDTA, 0.4% NP-40, and protease inhibitor cocktail) for 15 min at 4 °C. The homogenates were centrifuged for 30 s at 3000 ×*g* at 4 °C. The supernatant was removed. The pellets were lysed with 300 μL extraction buffer (20 mmol/L HEPES, pH 7.9, 0.4 mol/L NaCl, 1 mmol/L EDTA, and protease inhibitor

cocktail) for 30 min on a shaker set at 1000 rpm at 4 °C for nuclear protein isolation. Nuclear extracts were diluted with dilution buffer (20 mmol/L HEPES, pH 7.9, 1 mmol/L $MgCl_2$, 0.5% NP-40, 1 mmol/L EDTA, and protease inhibitor cocktail) at a ratio of 1:2, and incubated with either 50 μL Anti-HA-tag mAb-Magnetic Beads (MBL, M180-11) or 50 μL Mouse IgG1 (isotype control)-Magnetic Beads (MBL, M075-11) overnight at 4 °C. Immunoprecipitation beads were washed with wash buffer (50 mmol/L Tris-HCl, pH7.5, 200 mmol/L NaCl, 5 mmol/L EDTA, and 1% Triton X-100) for 3 times, and immunoprecipitated proteins were eluted by 1× protein loading buffer. The eluted proteins were then subjected to Western blotting analysis. The source data of the uncropped immunoblots are provided in the Source Data file.

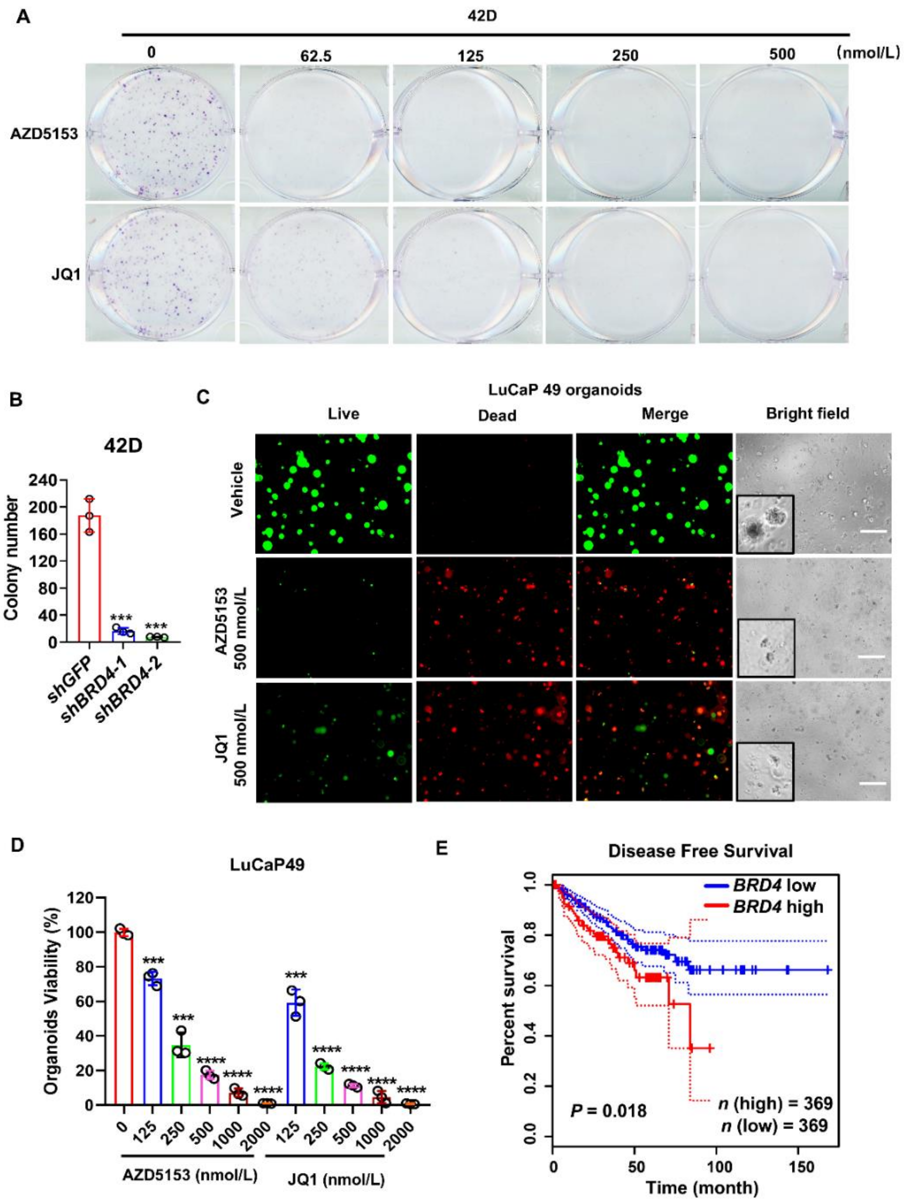

Figure S1 *BRD4* is overexpressed in NEPC tumors and required for NEPC cell growth and survival. (A) 42D cells were treated with indicated concentrations of BRD4 inhibitors JQ1 and AZD5153 for 14 days and then the photos of cell colonies were taken. (B) 42D cells were infected

with lentivirus expressing control shRNA against GFP or two different shRNAs against *BRD4* for 14 days and then cell colonies were counted. (C, D) LuCaP49 PDX-derived organoids were treated with DMSO or indicated concentrations of BRD4 inhibitors. Four days later, representative images were taken under a fluorescence microscope or standard light microscope. Organoid viability was measured using CellTiter-Glo. (E) Kaplan–Meier plot showing disease free survival over time of patients with *BRD4* high expressed tumors (red) *versus* low expressed tumors (blue) in GEPIA PRAD data set. Data are shown as mean ± SD; n=3. Student's t test. $^{**}P< 0.01$, $^{***}P<0.001$, $^{****}P<0.0001$.

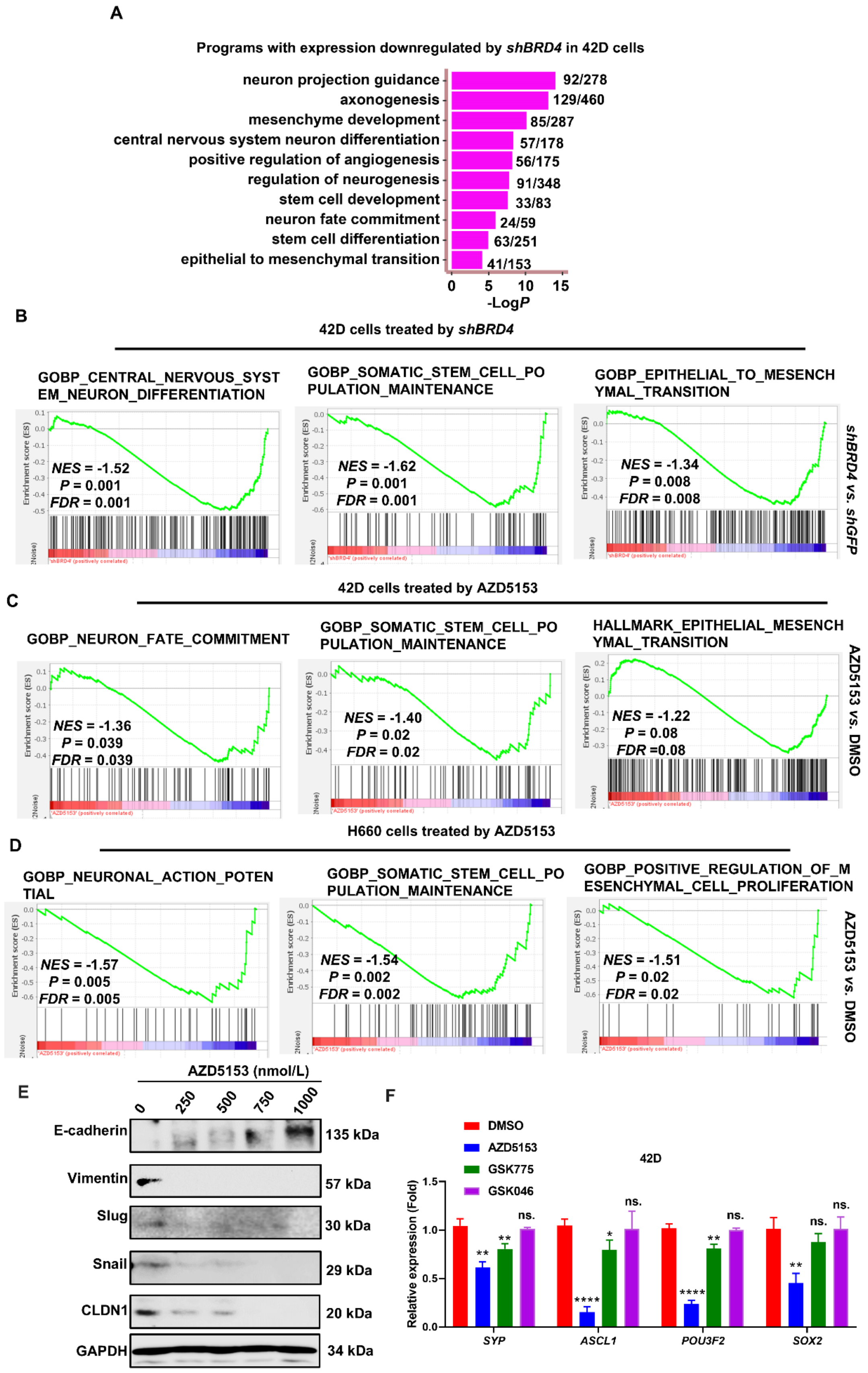
A
Programs with expression downregulated by shBRD4 in 42D cells
neuron projection guidance 92/278
axonogenesis 129/460
mesenchyme development 85/287
central nervous system neuron differentiation 57/178
positive regulation of angiogenesis 56/175
regulation of neurogenesis 91/348
stem cell development 33/83
neuron fate commitment 24/59
stem cell differentiation 63/251
epithelial to mesenchymal transition 41/153
-LogP
B
42D cells treated by shBRD4
GOBP_CENTRAL_NERVOUS_SYSTEM_NEURON_DIFFERENTIATION
NES = -1.52 P = 0.001 FDR = 0.001
GOBP_SOMATIC_STEM_CELL_POPULATION_MAINTENANCE
NES = -1.62 P = 0.001 FDR = 0.001
GOBP_EPITHELIAL_TO_MESENCHYMAL_TRANSITION
NES = -1.34 P = 0.008 FDR = 0.008
shBRD4 vs. shGFP
C
42D cells treated by AZD5153
GOBP_NEURON_FATE_COMMITMENT
NES = -1.36 P = 0.039 FDR = 0.039
GOBP_SOMATIC_STEM_CELL_POPULATION_MAINTENANCE
NES = -1.40 P = 0.02 FDR = 0.02
HALLMARK_EPITHELIAL_MESENCHYMAL_TRANSITION
NES = -1.22 P = 0.08 FDR =0.08
AZD5153 vs. DMSO
D
H660 cells treated by AZD5153
GOBP_NEURONAL_ACTION_POTENTIAL
NES = -1.57 P = 0.005 FDR = 0.005
GOBP_SOMATIC_STEM_CELL_POPULATION_MAINTENANCE
NES = -1.54 P = 0.002 FDR = 0.002
GOBP_POSITIVE_REGULATION_OF_MESENCHYMAL_CELL_PROLIFERATION
NES = -1.51 P = 0.02 FDR = 0.02
AZD5153 vs. DMSO
E
AZD5153 (nmol/L)
0 250 500 750 1000
E-cadherin 135 kDa
Vimentin 57 kDa
Slug 30 kDa
Snail 29 kDa
CLDN1 20 kDa
GAPDH 34 kDa
F
DMSO
AZD5153
GSK775
GSK046
42D
Relative expression (Fold)
SYP
ASCL1
POU3F2
SOX2

Figure S2 BRD4 controls lineage plasticity (LP) programs in NEPC. (A) Gene ontology (GO) analysis of protein-coding genes with expression significantly (>1.5-fold) downregulated by BRD4 knock down in 42D cells. Top 10 representative LP programs were shown. Also shown at right are the number of downregulated genes and total number of genes in each program. (B–D) GSEA plots depicting the significantly enrichment of genes in neurogenesis, stem cell and EMT in *BRD4* knocking down 42D cells (B) and 42D (C) and H660 (D) cells treated with BRD4 inhibitor AZD5153. (E) Immunoblotting of proteins involved in EMT in H660 cells treated with indicated concentrations of BRD4 inhibitors AZD5153 for 2 days. (F) qRT-PCR analysis of indicated gene expression in 42D cells treated with the bromodomain specific inhibitors. Data are shown as mean ± SD. Student's *t* test. $^{**}P< 0.01$, $^{***}P<0.001$, $^{****}P<0.0001$.

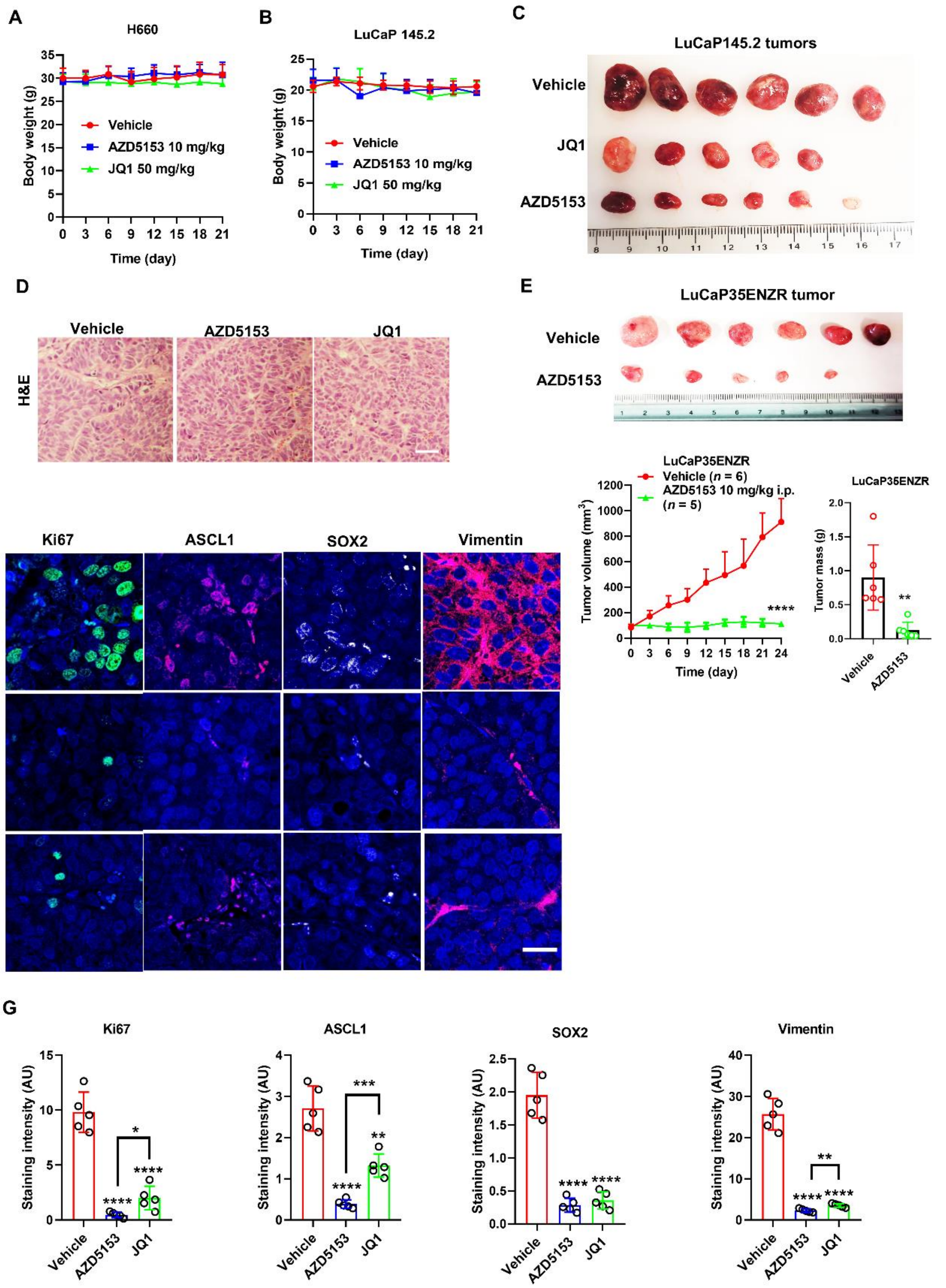

A
H660
Body weight (g)
Vehicle
AZD5153 10 mg/kg
JQ1 50 mg/kg
Time (day)
B
LuCaP 145.2
Body weight (g)
Vehicle
AZD5153 10 mg/kg
JQ1 50 mg/kg
Time (day)
C
LuCaP145.2 tumors
Vehicle
JQ1
AZD5153
D
Vehicle
AZD5153
JQ1
H&E
E
LuCaP35ENZR tumor
Vehicle
AZD5153
LuCaP35ENZR
Vehicle (n = 6)
AZD5153 10 mg/kg i.p. (n = 5)
Tumor volume (mm3)
Time (day)
****
LuCaP35ENZR
Tumor mass (g)
**
Vehicle
AZD5153
F
Ki67
ASCL1
SOX2
Vimentin
Vehicle
AZD5153
JQ1
G
Ki67
ASCL1
SOX2
Vimentin
Staining intensity (AU)
Vehicle
AZD5153
JQ1

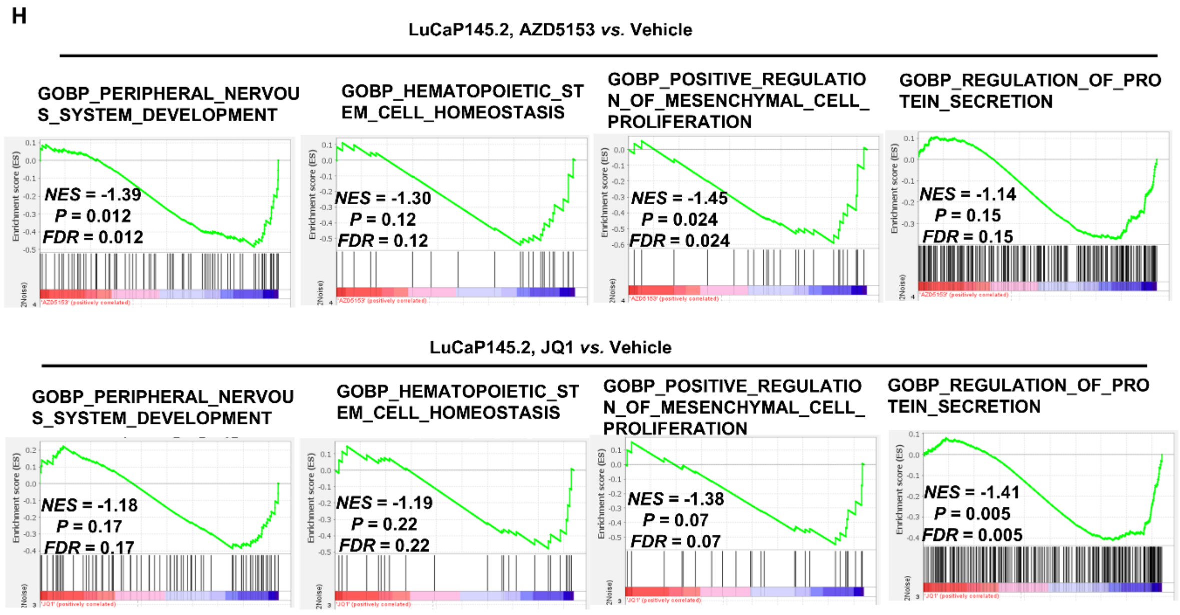

Figure S3 BRD4 inhibitors potently inhibited NEPC tumor growth and LP programs *in vivo*. (A, B) Mice bearing H660 and LuCaP145.2 tumors were treated, i.p., 5 times per week, with vehicle, 50 mg/kg JQ1 and 10 mg/kg AZD5153 for 21 days. Mouse bodyweight was measured every 3 days. (C) Photo images of LuCaP145.2 tumors dissected from mice treated with the inhibitors or vehicle. (D) Images of H&E staining of LuCaP145.2 tumors treated as above. Bar = 200 μm. (E) Mice bearing LuCaP35ENZR tumors were treated, i.p., 5 times per week, with vehicle, 50 mg/kg JQ1 and 10 mg/kg AZD5153 for 24 days in combination with 20 mg/kg enzalutamide (*p.o.*). Tumor volume was measured every 3 days and tumor growth curves were drawn to show growth of tumors of each group. Photo image of dissected tumors was shown. (F, G) Ki67, ASCL1, SOX2 and vimentin immunofluorescence were performed. Representative images from three independent tumors are shown. Staining intensity of these proteins was measured by image J using at least 5 random images. (H) GSEA plots depicting the significantly enrichment of genes in neurogenesis, stem cell, EMT and protein secretion in LuCaP145.2 tumors treated with BRD4 inhibitors AZD5153 and JQ1. Data are shown as mean ± SD. Student's *t* test. $^{**}P < 0.01$, $^{***}P<0.001$, $^{****}P<0.0001$.

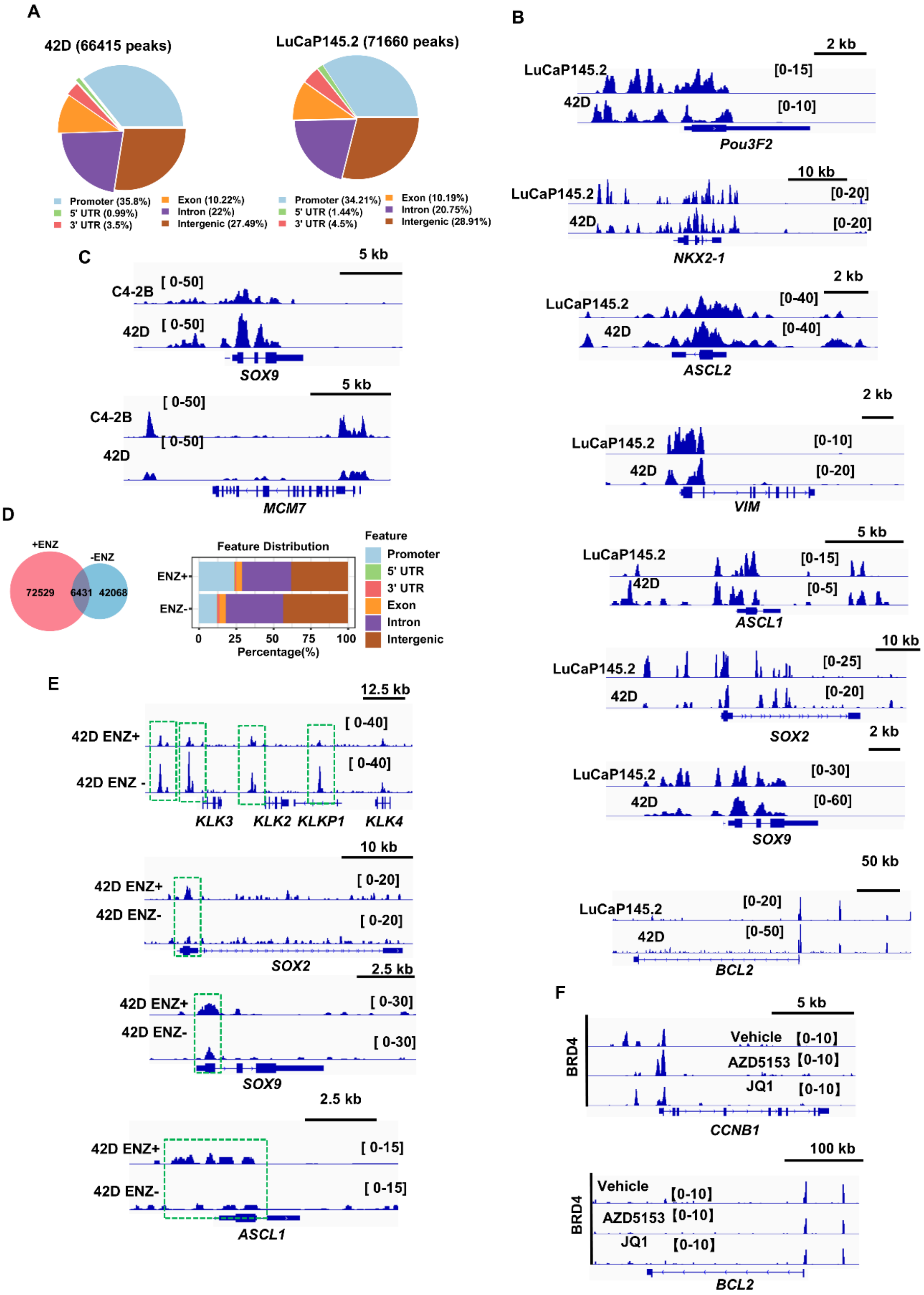

A
42D (66415 peaks)
LuCaP145.2 (71660 peaks)
Promoter (35.8%)
5' UTR (0.99%)
3' UTR (3.5%)
Exon (10.22%)
Intron (22%)
Intergenic (27.49%)
Promoter (34.21%)
5' UTR (1.44%)
3' UTR (4.5%)
Exon (10.19%)
Intron (20.75%)
Intergenic (28.91%)
B
2 kb
LuCaP145.2
[0-15]
42D
[0-10]
Pou3F2
10 kb
LuCaP145.2
[0-20]
42D
[0-20]
NKX2-1
2 kb
LuCaP145.2
[0-40]
42D
[0-40]
ASCL2
2 kb
LuCaP145.2
[0-10]
42D
[0-20]
VIM
5 kb
LuCaP145.2
[0-15]
42D
[0-5]
ASCL1
10 kb
LuCaP145.2
[0-25]
42D
[0-20]
SOX2
2 kb
LuCaP145.2
[0-30]
42D
[0-60]
SOX9
50 kb
LuCaP145.2
[0-20]
42D
[0-50]
BCL2
C
5 kb
C4-2B
[0-50]
42D
[0-50]
SOX9
5 kb
C4-2B
[0-50]
42D
[0-50]
MCM7
D
+ENZ
-ENZ
72529
6431
42068
Feature Distribution
ENZ+
ENZ-
0
25
50
75
100
Percentage(%)
Feature
Promoter
5' UTR
3' UTR
Exon
Intron
Intergenic
E
12.5 kb
42D ENZ+
[0-40]
42D ENZ -
[0-40]
KLK3
KLK2
KLKP1
KLK4
10 kb
42D ENZ+
[0-20]
42D ENZ-
[0-20]
SOX2
2.5 kb
42D ENZ+
[0-30]
42D ENZ-
[0-30]
SOX9
2.5 kb
42D ENZ+
[0-15]
42D ENZ-
[0-15]
ASCL1
F
5 kb
BRD4
Vehicle
[0-10]
AZD5153
[0-10]
JQ1
[0-10]
CCNB1
100 kb
BRD4
Vehicle
[0-10]
AZD5153
[0-10]
JQ1
[0-10]
BCL2

Figure S4 BRD4 is reprogrammed by ENZ and preferably direct control LP programs to both promoters and enhancers in NEPC. (A) Genomic distribution of BRD4 ChIP-seq peaks in 42D cells and LuCaP145.2 tumors. (B) IGV displays of BRD4 occupancy at LP genes *POU3F2*, *NKX2-1*, *ASCL2*, *VIM*, *ASCL1* and *SOX2* and E2F1-controlled gene *BCL2* in 42D cells and LuCaP145.2 tumors. (C) IGV displays of LP gene *SOX9* and cell cycle gene *MCM7* in adenocarcinoma C4-2B cells and t-NEPC 42D cells. (D) Left, Venn diagrams show the overlap of BRD4 bound sites with or without ENZ treatment. Right, the bar graphs show the genome wide distribution of BRD4 bound sites in 42D cells. (E) IGV snapshots of BRD4 occupancy at AR targets *KLKs* and LP drivers *SOX9*, *SOX2* and *ASCL1* in 42D cells with or without ENZ treatment. (F) IGV snapshots of BRD4 occupancy at cell cycle related genes *BCL2* and *CCNB1*. Also shown are the number of genes with BRD4 ChIP-seq peaks and the total gene number of each program.

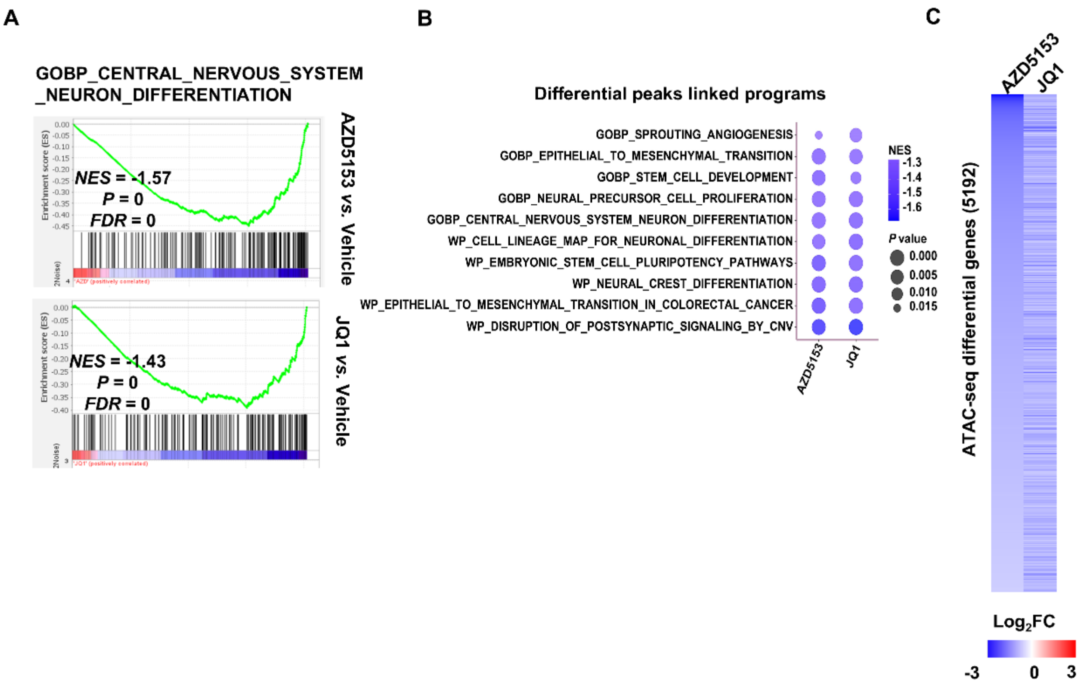

Figure S5 BRD4 stimulates LP programs through increasing chromatin accessibility. (A) GSEA analysis of decreased chromatin accessibility-linked LP programs in LuCaP145.2 tumors treated with JQ1 and AZD5153 *in vivo*. (B) Bubble plots depicting the significant enrichment of LP programs with chromatin accessibility in LP programs downregulated by BRD4 inhibitors. (C) Heatmaps display the $\log_2$ fold change of chromatin accessibility of genes commonly decreased by AZD5153 and JQ1 treatment as in Fig. 5B.

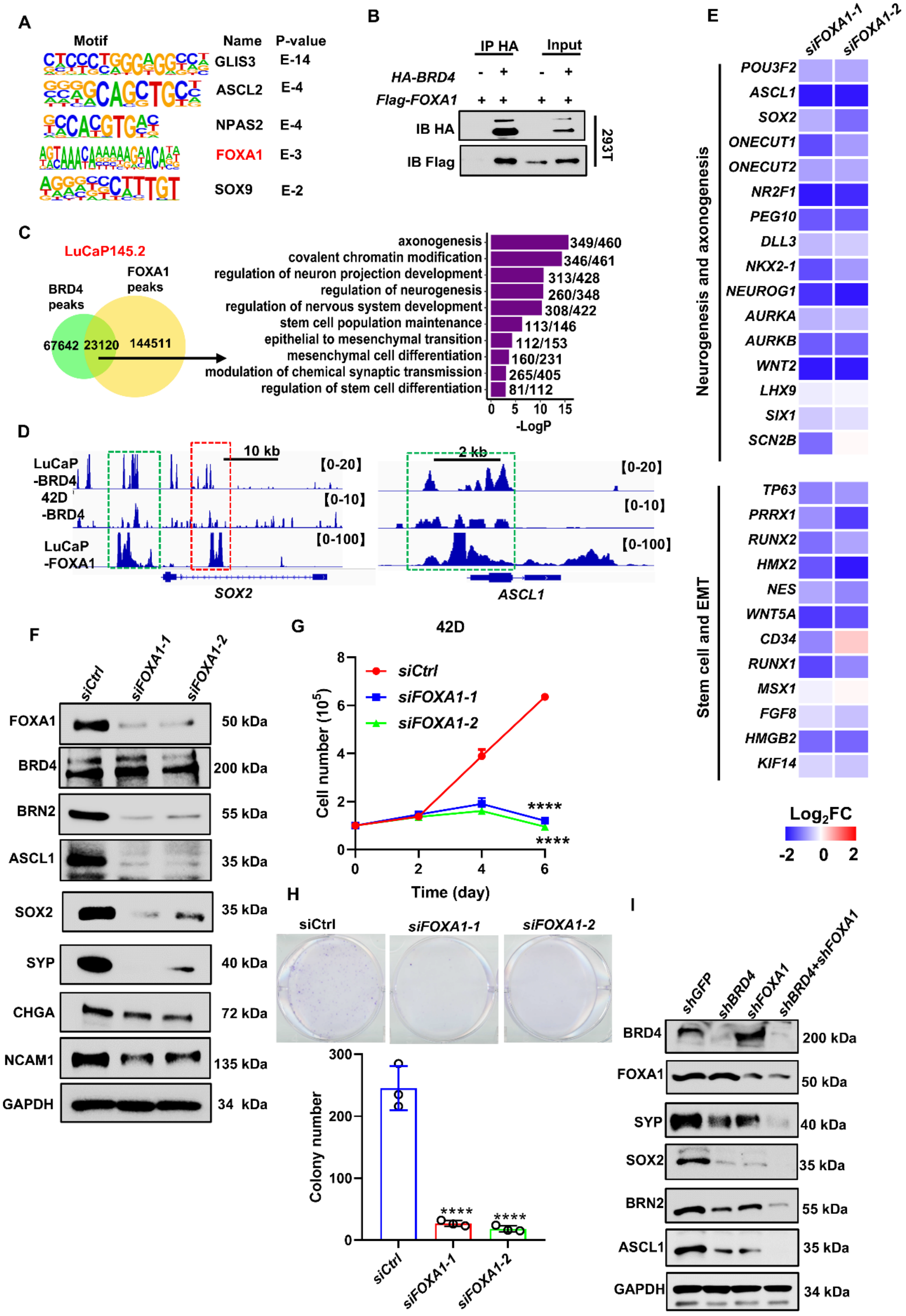

A
Motif
Name
P-value
GLIS3
E-14
ASCL2
E-4
NPAS2
E-4
FOXA1
E-3
SOX9
E-2
B
IP HA
Input
HA-BRD4
Flag-FOXA1
IB HA
IB Flag
293T
C
LuCaP145.2
BRD4 peaks
FOXA1 peaks
67642
23120
144511
axonogenesis
covalent chromatin modification
regulation of neuron projection development
regulation of neurogenesis
regulation of nervous system development
stem cell population maintenance
epithelial to mesenchymal transition
mesenchymal cell differentiation
modulation of chemical synaptic transmission
regulation of stem cell differentiation
349/460
346/461
313/428
260/348
308/422
113/146
112/153
160/231
265/405
81/112
-LogP
D
LuCaP -BRD4
42D -BRD4
LuCaP -FOXA1
10 kb
2 kb
[0-20]
[0-10]
[0-100]
SOX2
ASCL1
E
siFOXA1-1
siFOXA1-2
Neurogenesis and axonogenesis
POU3F2
ASCL1
SOX2
ONECUT1
ONECUT2
NR2F1
PEG10
DLL3
NKX2-1
NEUROG1
AURKA
AURKB
WNT2
LHX9
SIX1
SCN2B
Stem cell and EMT
TP63
PRRX1
RUNX2
HMX2
NES
WNT5A
CD34
RUNX1
MSX1
FGF8
HMGB2
KIF14
Log2FC
F
siCtrl
siFOXA1-1
siFOXA1-2
FOXA1
BRD4
BRN2
ASCL1
SOX2
SYP
CHGA
NCAM1
GAPDH
50 kDa
200 kDa
55 kDa
35 kDa
35 kDa
40 kDa
72 kDa
135 kDa
34 kDa
G
42D
siCtrl
siFOXA1-1
siFOXA1-2
Cell number (10^5)
Time (day)
****
****
H
siCtrl
siFOXA1-1
siFOXA1-2
Colony number
****
****
I
shGFP
shBRD4
shFOXA1
shBRD4+shFOXA1
BRD4
FOXA1
SYP
SOX2
BRN2
ASCL1
GAPDH
200 kDa
50 kDa
40 kDa
35 kDa
55 kDa
35 kDa
34 kDa

Figure S6 FOXA1 facilitates BRD4 recruitment to the LP gene targets. (A) Motif enrichment analysis of BRD4 decreased ChIP-seq peaks induced by AZD5153 treatment in LuCaP145.2 tumors, listing 5 representative TFs linked to LP programs. (B) 42D cells were co-transfected with vectors that expressing HA-BRD4 and Flag-FOXA1. The nuclear extracts were used for co-IP with indicated antibodies. The experiments were repeated three times. (C) Venn diagram shows the overlapped ChIP-seq peaks of BRD4 and FOXA1 in LuCaP145.2 tumors. The overlapped peaks linked genes were subjected to Gene ontology (GO) analysis and top 10 representative programs are shown. (D) IGV snapshots of BRD4 and FOXA1 chromatin occupancy at LP drivers *SOX2* and *ASCL1*. (E) Heatmaps show the genes in LP programs including Neurogenesis, stem cell and EMT that are downregulated by *FOXA1* knockdown in 42D cells. (F) 42D cells were infected with lentivirus containing siRNAs targeting FOXA1 for 72 h. The expression of BRD4 and NEPC drivers BRN2, ASCL1, SOX2 and NEPC marker SYP was detected by immunoblotting. (G) Growth curve of 42D cells that infected with lentivirus containing siRNAs targeting FOXA1 were plotted. (H) 42D cells were transfected with siRNAs targeting *FOXA1* for 14 days and then cell colonies were counted. (I) 42D cells were infected with lentivirus containing shRNAs targeting *BRD4* and *FOXA1* for 72 h, before cell lysis was harvested for the detecting of the expression of proteins in LP. Data are shown as mean ± SD; $n = 3$. Student's t test. $^{**}P < 0.01$, $^{***}P<0.001$, $^{****}P<0.0001$.

Table S1 Antibodies used in this study.

Antibody names	Product code	Brand	Application
Anti-BRD4	54615S	Cell Signaling Technology	WB, 1:1000
Anti-BRN2	12137S	Cell Signaling Technology	WB, 1:1000 IF, 1:200
Anti-ASCL1	ab211327	Abcam	WB, 1:1000 IF, 1:200
Anti-SOX2	92494	Abcam	WB, 1:1000 IF, 1:200
Anti-SYP	36406S	Cell Signaling Technology	WB, 1:1000 IF, 1:200
Anti-CHGA	ab283265	Abcam	WB, 1:1000
Anti-ENO2	65162S	Cell Signaling Technology	WB, 1:1000
Anti-NCAM1	99746S	Cell Signaling Technology	WB, 1:1000
Anti-GAPDH	2118L	Cell Signaling Technology	WB, 1:1000
Anti-PARP	5625S	Cell Signaling Technology	WB, 1:1000
Anti-Cleaved Caspase3	9664S	Cell Signaling Technology	WB, 1:1000
Anti-Ecadherin	3195S	Cell Signaling Technology	WB, 1:1000
Anti-CLDN1	13255S	Cell Signaling Technology	WB, 1:1000
Anti-Snail	3879S	Cell Signaling Technology	WB, 1:1000
Anti-Slug	9585S	Cell Signaling Technology	WB, 1:1000
Anti-Flag	ab205606	Abcam	IP, 10 μg per IP
anti-Vimentin	5741S	Cell Signaling Technology	WB, 1:1000 IF, 1:200
anti-Ki67	ab15580	Abcam	IF, 1:200
anti-FOXA1	ab170933	Abcam	WB, 1:1000
Goat anti-Rabbit IgG HRP	1721019	Bio-Rad	WB, 1:2000
Goat anti-Mouse IgG HRP	1721011	Bio-Rad	WB, 1:2000
Anti-BRD4	Diagenode	C15410337	ChIP-seq, 4 μg per IP
Anti-H3K27ac	Diagenode	C15410196	ChIP-seq, 4 μg per IP

Table S2 qPCR primer sequences.

Gene symbol	Forword primer 5′–3′	Reverse primer 5′–3′
POU3F2 (BRN2)	ACACTGACCGATCTCCACGCAGTA	GAGGGTGTGGGACCCTAAATATGAC
ASCL1	CGCGGCCAACAAGAAGATG	CGACGAGTAGGATGAGACCG
SOX2	TGCGAGCGCTGCACA	TCATGAGCGTCTTGGTTTTCC
SYP	TCAGTTCCGGGTGGTCAAG	AAGACCCATTGCAGCACCTT
GAPDH	CGACCTGACCTGCCGTCTAGAA	GGTGTCGCTGGTGAAGTCGAGAG

DatasetS01 Tables S3-S11

Table S3: Gene expression derived from RNA sequencing data of BRD4 and FOXA1 knockdown in 42D cells (FPKM)

Table S4: Gene expression derived from RNA sequencing data of 42D cells treated with BRD4 inhibtors for 48 h (FPKM)

Table S5: Gene expression derived from RNA sequencing data of H660 cells treated with BRD4 inhibtors for 48 h (FPKM)

Table S6: Gene expression derived from RNA sequencing data of LuCaP145.2 treated with BRD4 inhibtors for 7 days (FPKM)

TableS7: GO analysis of genes downregulated by shBRD4 for 48 h (log2FC>1.5)

TableS8: GO analysis of genes commonly downregulated by AZD5153 and JQ1 in 42D cells (log2FC>1.5)

TableS9: GO analysis of genes commonly downregulated by AZD5153 and JQ1 in H660 cells (log2FC>1.5)

TableS10: GO analysis of genes downregulated by AZD5153 in LuCaP145.2 tumors (log2FC>1.5)

Table11: GO analysis of genes downregulated by JQ1 in LuCaP145.2 tumors (log2FC>1.5)

DatasetS02 Tables S12-S16

Table 12: Manorm normalized ATAC-seq peak intensity (Vehicle and AZD5153)

Table 13: Manorm normalized ATAC-seq peak intensity (Vehicle and JQ1)

TableS14: Differential ATAC-seq peaks identifed by Manorm analysis

TableS15: GSEA analysis of ATAC-seq peak intensity identifed by Manorm (AZD5153 vs. Vehicle) in LuCaP145.2 tumor

TableS16: GSEA analysis of ATAC-seq peak intensity identifed by Manorm (JQ1 vs. Vehicle) in LuCaP145.2 tumor

DatasetS03 Tables S17-S26

TableS17: BRD4-bound peaks in 42D

TableS18: BRD4-bound peaks in LuCaP145.2

TableS19: BRD4-bound peaks-linked programs in 42D cells

TableS20: BRD4-bound peaks-linked programs in LuCaP145.2 tumors

TableS21: BRD4-bound peaks in C4-2B

TableS22: BRD4-bound peaks-linked programs in C4-2B

TableS23: BRD4 and FOXA1 co-bound peaks-linked programs in LuCaP145.2

TableS24: BRD4 and FOXA1 co-bound peaks in LuCaP145.2

TableS25 BRD4 and FOXA1 co-bound peaks density normalized by Manorm

TableS26 GSEA analysis of reduced BRD4 peaks enriched programs after FOXA1 knockdown

DatasetS04 Tables S27-S28

TableS27: Homer analysis of BRD4 ChIP-seq lost peaks treated by AZD5153 in LuCaP145.2 tumors

TableS28: Homer analysis of ATAC-seq lost peaks treated by AZD5153 in LuCaP145.2 tumors
